# Artificial intelligence-driven drug discovery: a deep learning paradigm shift in pharmaceutical research and development

**DOI:** 10.3389/fphar.2026.1927954

**Published:** 2026-09-09

**Authors:** Xueyuan Bi, Yangyang Wang, Jihan Wang, Cuicui Liu

**Affiliations:** 1 Department of Pharmacy, Honghui Hospital, Xi’an Jiaotong University, Xi’an, China; 2 School of Physics and Electronic Information, Yan’an University, Yan’an, Shaanxi, China; 3 Yan’an Medical College of Yan’an University, Yan’an, Shaanxi, China; 4 Department of Science and Education, Honghui Hospital, Xi’an Jiaotong University, Xi’an, China

**Keywords:** ADMET, chemical space, *de novo* molecular design, deep learning, drug discovery, generative AI

## Abstract

Efficient drug discovery is essential to mitigate the high attrition rates and capital-intensive nature of traditional pharmaceutical research. Deep learning (DL) has catalyzed a paradigm shift, offering unprecedented computational capabilities to accelerate this process. This review systematically summarizes advancements in DL methodologies for drug discovery. First, the critical data foundations, Molecular Representation Learning, and core architectures underpinning artificial intelligence (AI)-driven therapeutics are elucidated, with a particular emphasis on the evolution of generative AI frameworks (such as Variational Autoencoders (VAEs), Generative Adversarial Networks (GANs), and diffusion models) for chemical space exploration. Subsequently, diverse DL applications across the discovery pipeline are investigated, encompassing target identification, virtual screening, *de novo* molecular design, retrosynthetic analysis, absorption, distribution, metabolism, excretion, and toxicity (ADMET) profiling, and drug repurposing. Furthermore, persistent challenges, particularly data scarcity, model opacity, experimental validation bottlenecks, and escalating computational resource requirements, are critically evaluated. To bridge the gap between *in silico* predictions, chemical synthesis, and biological reality, emerging solutions such as federated learning, causality-aware Explainable AI (XAI), closed-loop autonomous laboratories, and efficiency-oriented algorithmic strategies are explored. Ultimately, this article provides strategic insights into leveraging DL, underscoring its transformative potential in driving next-generation medicinal and therapeutic innovation.

## Graphical Abstract

**Figure:**
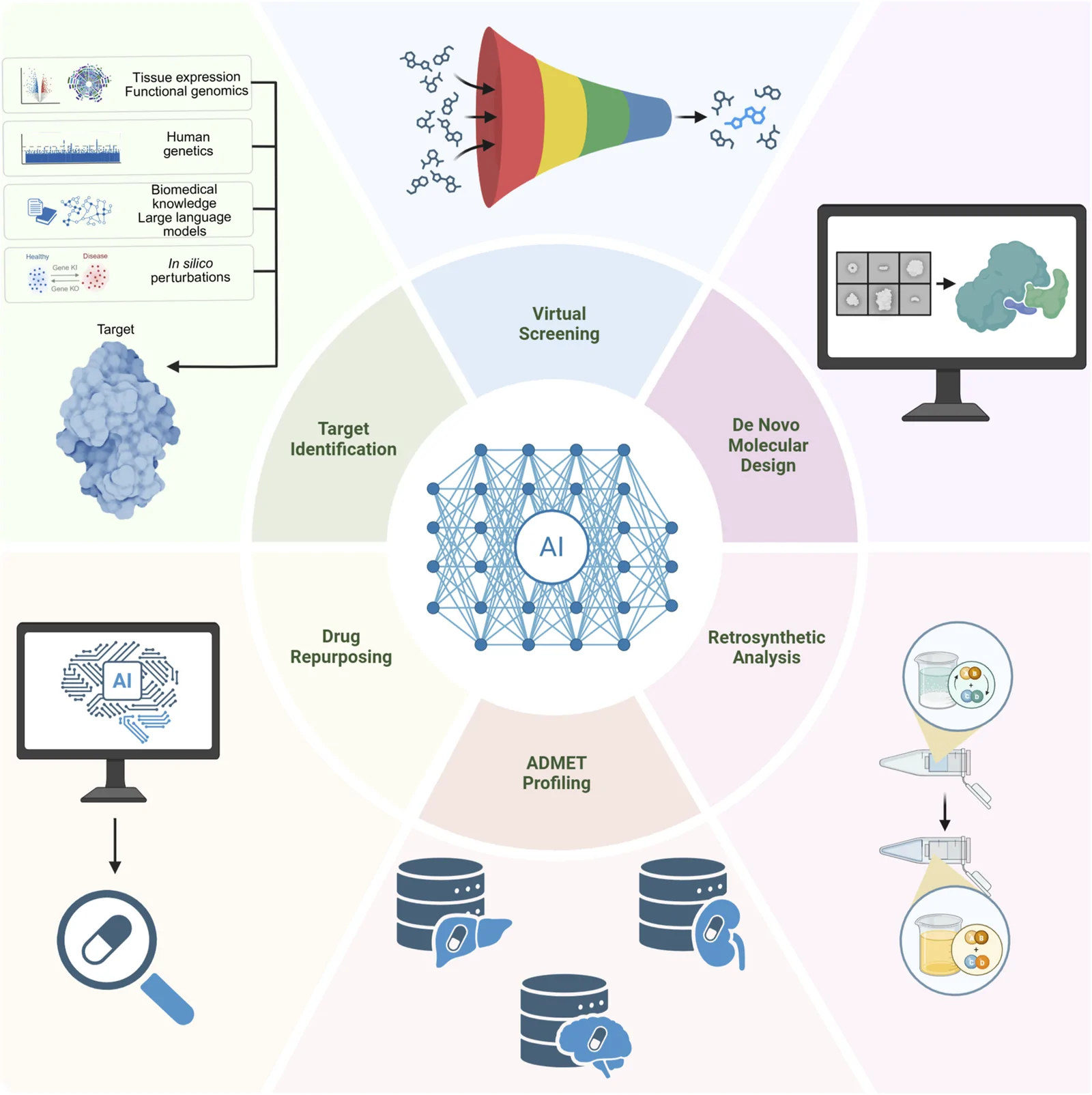

## 1 Introduction

Traditional pharmaceutical research and drug development are notoriously complex, resource-intensive, and inherently risky endeavors (Nag et al., 2022; Mak et al., 2024). The established pipeline typically spans over a decade, requires immense capital investment, and frequently suffers from high attrition rates during late-stage clinical trials (Zhou et al., 2025). A significant portion of these failures can be attributed to unforeseen toxicity or lack of clinical efficacy, highlighting the persistent difficulty in predicting how novel compounds will behave within the human body (Zhang J. et al., 2025). Because of these systemic limitations, establishing more efficient and predictive drug discovery frameworks is essential to mitigate the significant financial burdens and extended timelines associated with bringing a new biological entity to market (Noor et al., 2024). Historically, computational methods in pharmacology relied on manual feature extraction and simplified physical models. While these early approaches provided a foundation for the field, they often failed to capture the immense, non-linear complexity of biological systems (Jiang et al., 2021).

The advent of the big data era across the biological and chemical sciences has necessitated a strategic move away from traditional, human-centric feature engineering toward more autonomous and robust data-driven methodologies (Moon et al., 2022; Atz et al., 2024; Yu et al., 2024). Recently, deep learning (DL) has catalyzed a fundamental paradigm shift in the pharmaceutical sciences, offering unprecedented computational capabilities to accelerate and systematically optimize the entire drug discovery and development pipeline from inception to clinical evaluation (Born and Manica, 2021; Liu et al., 2021; Wang K. et al., 2021). Unlike conventional statistical algorithms, DL models thrive on the massive, heterogeneous datasets generated by modern high-throughput screening and comprehensive biological profiling techniques. By effectively processing large-scale biological and chemical datasets, these algorithms excel at uncovering hidden patterns and high-order correlations—such as intricate structure-activity relationships and latent therapeutic vulnerabilities—that elude traditional analytical methods (Peng et al., 2022; Wu et al., 2022; Gao et al., 2024). This shift replaces isolated tasks with a cohesive AI engine for navigating chemical and biological landscapes.

Building upon this robust data-driven ecosystem, specific DL architectures have been tailored to address distinct translational challenges across the drug discovery continuum. Advanced computational architectures, specifically Graph Neural Networks, Variational Autoencoders, Transformers, and Convolutional Neural Networks, among others, have become highly effective at extracting robust, hierarchical molecular representations from both complex biological networks and raw chemical structures (Ahmed et al., 2021; Withnell et al., 2021; Chen et al., 2025a). This enhanced computational power is particularly critical for navigating the extraordinarily vast and sparse chemical space, which encompasses a virtually limitless array of potentially drug-like molecules, thereby significantly accelerating the entire developmental pipeline. In practice, these sophisticated models facilitate precise target identification and validation by integrating multi-source heterogeneous data, streamline virtual screening for hit discovery, and guide the *de novo* generation of optimized lead compounds with superior pharmacological profiles (Atance et al., 2022; Su et al., 2023; Blaudin De Thé et al., 2025). Furthermore, they significantly enhance reaction prediction for retrosynthetic analysis, improve the accuracy of comprehensive ADMET and toxicity profiling, and unlock novel therapeutic avenues through intelligent drug repurposing (Yao et al., 2023; Swanson et al., 2024; Liu et al., 2026). By moving beyond human-centric feature engineering, these methodologies provide an autonomous and data-driven framework for drug discovery.

Recent reviews on AI-driven drug discovery have largely remained either task-oriented or implementation-focused. For example, Hu et al. emphasized molecular generation, property prediction (Hu et al., 2024), retrosynthesis, and reaction prediction, primarily from the perspective of benchmarks and model taxonomies, while offering limited discussion of translational deployment and multimodal integration. Ferreira and Carneiro provided a broader pipeline-level overview centered on implementation strategies (Ferreira and Carneiro, 2025), validation, and ethical considerations, but placed less emphasis on systematically connecting molecular representation learning, architectural evolution, and translational bottlenecks. Despite these advances, the field still lacks an integrative account of how data foundations and molecular representations determine the capabilities and limitations of specific deep learning architectures, how these architectural trade-offs should guide model selection for different pharmaceutical tasks, and how computational performance should be interpreted in relation to chemical validity, experimental evidence, and translational feasibility. The present review addresses this gap by organizing the evidence along a continuous representation–architecture–application–translation framework rather than treating algorithms or drug-discovery tasks as isolated topics. A distinctive contribution of this review is the incorporation of validation as an explicit comparative dimension: Table 1 summarizes the validation strategy reported in each representative study and critically evaluates its strengths and limitations, thereby clarifying the reliability and practical relevance of the reported findings.

**TABLE 1:** Summary of applications and case studies utilizing DL for drug discovery.

Task domain	Model/approach	Dataset	Key findings and metrics	Validation strategy	Validation strengths and limitations	References
Target identification	MOGOLA (Graph-based DL)	Datasets from AD Knowledge Portal and GDC Data Portal	Achieved end-to-end integration without manual feature selection; identified hub genes CCNA2, CDK1, and ESR1; supported HER2-directed subtype-specific therapy	Benchmark evaluation on BRCA, KIPAN, ROSMAP, and LGG against 11 methods; ablation analyses; functional-enrichment and PPI-based biomarker assessment	Multi-dataset benchmarking supports computational robustness, though further architectural module optimization and increased resilience to data heterogeneity are recognized for future expansion	Huang et al. (2025)
	NETTAG (Interpretable GNN framework)	GWAS Catalog, GTEx	Predicted 156 AD-related genes; prioritized 9 core targets (e.g., BACE1, GSK3B, FYN); linked specific drivers to amyloid-β and tau pathology; facilitated drug repurposing	ROC/AUC evaluation using four AD-associated gene sets; GWAS, transcriptomic, proteomic, and single-nucleus RNA-seq corroboration; retrospective EHR case–control analysis in 10 million individuals	Multi-level omics evidence and large-scale real-world data strengthen biological and clinical relevance. However, incomplete interactome and GWAS data may affect prediction, while observational EHR associations cannot establish therapeutic causality	Xu et al. (2022)
	Structure-based DL (integrating protein dynamics, accessibility, and mutability)	GISAID database (version March 2023) and UniProt (ID: P0DTC2)	Identified an allosteric binding site specific to both conformational and oligomeric states on the SARS-CoV-2 Spike protein; demonstrated that targeting this site shifts the equilibrium toward an inactive conformation to prevent ACE2 interaction; discovered hit compounds exhibiting viral inhibition at micromolar concentrations	Binding-site conservation and network analyses; molecular docking; molecular dynamics simulations; *in vitro* SARS-CoV-2 pseudotyped-virus assay	Comprehensive validation integrated structural analysis, virtual screening, molecular dynamics, cytotoxicity assays, and a pseudotyped-virus assay with a VSV-G control. However, potential docking artifacts, brief 100-ns MD simulations, and the absence of crystallographic/cryo-EM proof limit mechanistic certainty	Popov et al. (2023)
​	Deep autoencoder-based framework	Datasets from DrugBank databaseetc.	Prioritized novel putative therapeutic target genes (e.g., DLG4, EGFR, RAC1, PTK2B) for Alzheimer’s disease; successfully inferred promising repositionable candidate drugs including tamoxifen, bosutinib, and dasatinib	Fivefold cross-validation; pathway-enrichment analysis; literature-based biological corroboration of prioritized targets and drugs	Cross-validation and consistency with established AD mechanisms support hypothesis generation. However, the mean ROC-AUC was modest (0.661), and the predicted targets and repositioned drugs received no new experimental validation	Tsuji et al. (2021)
	OptiSyn (Interpretable multi-layer GCN framework)	Multi-source databases (TCMSP, DrugBank, PubChem, AlphaFold, GEO, etc.)	Identified 8 ankylosing spondylitis-associated hub genes; prioritized a synergistic therapeutic formula (ASD-A); experimentally validated *in vivo* and *in vitro* that ASD-A reduces pro-inflammatory cytokines, modulates immune cell subsets, and rebalances the IL17/Foxp3 axis by regulating core targets like KRAS, SMAD2, and MAPK14	Threefold cross-validation with multiple random seeds; seven-model benchmarking and ablation studies; independent GEO validation; *in vitro* Jurkat-cell and *in vivo* SKG-mouse experiments	Computational, external-dataset, cellular, and animal evidence provides comparatively strong support. Incomplete herbal databases, limited mechanistic depth, and a lack of clinical trial evidence restrict generalizability beyond the tested AS models	Shi et al. (2026)
Virtual screening	VirtuDockDL (GNN-based platform integrating ligand- and structure-based screening)	Datasets from Binding DB, PubChem, ChEMBL, and DUD-E; benchmarked on HER2 dataset	Developed an automated pipeline for high-throughput screening and protein refinement; achieved 99% accuracy and 0.99 AUC on HER2 dataset, surpassing DeepChem and AutoDock Vina; identified novel non-covalent inhibitors against the VP35 protein of Marburg virus	Test-set evaluation on HER2, TEM-1 beta-lactamase, and CYP51 datasets; comparisons with DeepChem, AutoDock Vina, Glide, PyRMD, RosettaVS, and MzDOCK; molecular-docking analyses and VP35 virtual screening	Validation combined classification metrics across multiple targets, benchmark comparisons with ML and docking tools, and a VP35-targeted screening case study. The predicted hits received no prospective *in vitro* or *in vivo* experimental validation	Noor et al. (2024)
​	PharmacoNet (DL-guided pharmacophore modeling)	Datasets from PDBbind v2020, ZINC20, ChEMBLetc.	Automated protein-based pharmacophore extraction; evaluated ligand potency via a parameterized analytical scoring function; ensured exceptional generalization across unseen targets and novel ligands for rapid screening	Virtual-screening benchmarks on DUD-E and DEKOIS2.0; runtime benchmarking on 363 PDBbind v2020 complexes; comparisons with docking and DL methods; ablation and restricted-data tests; one-million-compound KRAS-G12C pre-screening followed by SMINA.	Validation evaluated screening accuracy, runtime speed, out-of-distribution generalization, and an ultra-large-scale pre-screening case study. Its pharmacophore-level abstraction omits atom-level features, subtle salt-bridge/charge variations, and intramolecular energy dynamics	Seo and Kim (2024)
MolTrans (interaction transformer framework)	MolTrans (interaction transformer framework)	Datasets from PDBbind v2020, BIOSNAP, DAVIS, BindingDB, ZINC20, and ChEMBL	Demonstrated predictive robustness for drug-target interactions across four primary druggable categories; benchmarked against 1,908 enzyme, 533 GPCR, 496 ion channel, and 104 nuclear receptor interactions	Five random runs on BIOSNAP, DAVIS, and BindingDB with baseline comparisons; unseen-drug and unseen-protein splits; scarce-data tests; protein-family analysis; interpretability analysis and component/data-size ablations	Validation covered three datasets, unseen-drug/protein splits, scarce-data tests, multiple protein families, interpretability, and ablations. Relying exclusively on 1D molecular sequences omits 3D conformational dynamics and lacks prospective wet-lab confirmation	Huang et al. (2021)
	DeepRNA-DTI (Sequence-based architecture for RNA targets)	Datasets from PDB database, ROBIN dataset, RSApre datasetetc.	Demonstrated robust generalization across diverse RNA subtypes and was successfully applied to a high-throughput virtual screening of over 48 million compounds against oncogenic pre-miR-21	Benchmark and ablation evaluation of interaction and binding-site prediction across unseen-data settings; screening of over 48 million compounds against pre-miR-21	Validation assessed interaction prediction, binding-site localization, multiple generalization settings, RNA subtypes, model components, and large-scale screening. Point predictions and limited RNA–compound data constrain reliability and generalizability	Bae and Nam (2025)
​	Neural network (DL-guided virtual screening)	Datasets from GenBank and GEO	Discovered abaucin, a novel narrow-spectrum antibacterial compound against A. baumannii; elucidated its mechanism of action as perturbing lipoprotein trafficking via LolE; successfully validated its therapeutic efficacy in a murine wound infection model	*In vitro* screening (∼7,500 molecules); experimental testing; antibacterial-spectrum and clinical assays; resistance, transcriptomic, and mechanistic analyses; mouse wound evaluation	Model-ranked predictions were followed by compound testing, antibacterial-spectrum and mechanistic studies, and *in vivo* efficacy evaluation. Mechanistic interpretation partly relied on the better-studied *E. coli* system due to limited characterization of the A. baumannii Lol pathway	Liu G. et al. (2023)
	D3AI-CoV (Integrating MultiDTI, MPNNs-CNN, and MPNNs-CNN-R DL models)	Dataset of 842 molecules across 9 pathogenic coronaviruses and external Test-78 dataset.	Developed a platform for target prediction and virtual screening for the discovery of anti-COVID-19 drugs; achieved superior target prediction accuracy with AUCs of 0.93 (MultiDTI) and 0.91 (MPNNs-CNN); demonstrated higher virtual screening hit rates for anti-COVID-19 candidates compared to conventional methods	Tenfold cross-validation; independent Test-78 evaluation using 39 active and 39 inactive compounds; comparisons with existing target-prediction and virtual-screening webservers; hit-rate evaluation for 3C-like and papain-like proteases	Validation combined internal tenfold cross-validation, an independent Test-78 evaluation, webserver comparisons, and target-specific hit-rate assessments. Dependence on known interaction data limits out-of-distribution prediction, and screening results lack direct experimental structural validation	Yang et al. (2022)
*De Novo* molecular design and lead optimization	CNSMolGen (A bidirectional RNN-based generative model utilizing transfer learning for CNS-specific design)	ChEMBL-MPO (412,154 filtered molecules) and ChEMBL-ACT (271,914 bioactive compounds)	Generated >90% novel, synthesizable structures possessing optimal CNS drug properties; successfully fine-tuned for the serotonin transporter (SERT); validated potential bioactivity through physics-based induced-fit docking studies	Molecular validity, novelty, CNS properties, and synthetic accessibility evaluation; SERT-specific transfer learning and induced-fit docking	Evaluated both generative quality and predicted SERT binding. Target-specific validation relied on induced-fit docking, whose inherent limitations were acknowledged; fine-tuning used 36 SERT ligands	Gou et al. (2024)
​	DL-based generative models coupled with QSAR-based MPO scoring	Proprietary lead-optimization dataset (n = 881; 11 bioactivity, selectivity, and ADME endpoints)	Developed QSAR models with 0.67–1.0 precision; generated 150 virtual compounds predicted as active across all objectives; 11 candidates were synthesized, achieving an 86% success rate (9.5/11 objectives met) compared to 58% (6.4/11 objectives) for initial molecules; identified one molecule satisfying all 11 objectives and two meeting 10 objectives within error margins	QSAR-guided multiparameter optimization followed by synthesis and experimental testing of 11 AI-designed compounds across 11 objectives	Prospective synthesis and multi-endpoint assays directly evaluated generated compounds. Experimental validation comprised 11 compounds from a single lead-optimization project	Perron et al. (2022)
	DMDiff (Geometric spatial perception diffusion model)	The Crossdocked2020 dataset	Captured precise 3D spatial information to design molecules with high binding affinities; enabled target-specific molecular generation by leveraging geometric spatial perception	CrossDocked2020 benchmark comparisons using Vina-based affinity, molecular-property, structural-stability, interpretability, and ablation analyses	Evaluated affinity and drug-like properties on 100 new pockets against multiple baselines. The scoring functions may neglect solvation, entropy, induced fit, and protein dynamics	Lu H. et al. (2025)
	PMDM: conditional dual equivariant diffusion model (incorporating local and global dynamics)	Datasets from CrossDocked2020, PDBbind v2017	Outperformed baseline models in target-conditioned 3D molecule generation; successfully applied to real-world lead optimization for SARS-CoV-2 main protease (Mpro) and CDK2; experimentally validated synthesized candidates *in vitro*, demonstrating improved inhibitory activity against CDK2	CrossDocked benchmarking; docking, MM-PBSA, chemical-space analyses, synthesis, and CDK1/CDK2 enzyme assays	Combined benchmark evaluation with synthesis and *in vitro* activity testing. PMDM showed limited diversity in three-dimensional chemical space	Huang L. et al. (2024)
​	DiffLinker (E(3)-equivariant 3D-conditional diffusion model)	Datasets from ZINC, CASF, GEOM, and Pocketsetc.	Linked an arbitrary number of disconnected fragments while automatically determining linker sizes and attachment points; outperformed existing methods by generating more diverse and synthetically accessible molecules; successfully generated valid linkers conditioned on 3D target protein pockets in real-world applications	Four-dataset benchmarking, steric-clash and docking analyses, plus Hsp90, IMPDH, and JNK case studies	Covered pairwise, multi-fragment, and pocket-conditioned linker design. Post-generation bond assignment reduced validity; synthetic accessibility required improvement, and PROTAC-like linkers remained challenging	Igashov et al. (2024)
Reaction prediction and retrosynthetic analysis	G2Retro (Two-step graph generative framework; semi-template-based)	USPTO-50K	Imitates reversed logic via reaction center prediction and synthon completion; achieved 63.1% (known type) and 53.9% (unknown type) Top-1 accuracy on USPTO-50K; correctly predicted synthesis routes for Mitapivat (amide coupling) and Tapinarof (deprotection)	USPTO-50K top-k benchmarking with known and unknown reaction classes; module comparisons and case studies	Evaluated reaction-center identification and complete reactant generation. Does not cover every reaction-center type or unseen center-attached structures and requires reliable atom mapping	Chen et al. (2023)
	LocalRetro (Locally encoded retrosynthesis model with global attention)	USPTO-50K and USPTO-MIT (479,035 reactions)	Motivated by local atom/bond edits; utilized global attention to account for nonlocal functional group effects; achieved 89.5% Top-1 and 99.2% Top-5 round-trip accuracy on USPTO-50K; correctly predicted synthesis pathways for five drug candidate molecules	Top-k and round-trip evaluation on USPTO-50K and USPTO-MIT; attention analysis and five drug-synthesis case studies	Validated on two dataset scales and literature-reported multistep routes. In one drug case, the Suzuki coupling step was not predicted with high confidence	Chen and Jung (2021)
​	RetroPrime (Two-stage Transformer-based template-free method)	USPTO-50K, USPTO-full	Achieved Top-1 accuracy on USPTO-50K of 64.8% (reaction type known) and 51.4% (reaction type unknown). Achieved Top-1 accuracy of 44.1% on USPTO-full. Specifically designed to improve diversity and chemical plausibility over standard Transformer models	Top-k benchmarking on USPTO-50K and USPTO-full; output validity, diversity, and chemical-space generalization analyses	Evaluated accuracy, plausibility, diversity, and large-dataset generalization. Relies on high-quality atom mapping to extract reaction centers, and the fusion approach for merging predictions from multiple synthons is not achieved in a learnable way	Wang X. et al. (2021)
	RadicalRetro (Chemformer-based architecture utilizing a three-stage strategy)	RadicalDB (21.6K radical reactions); pretrained on ZINC-15 and USPTO	Achieved 69.3% Top-1 and 80.1% Top-10 accuracy, surpassing SOTA models by over 23%; effectively captures complex radical patterns like cascade cyclizations; proposed synthetically viable routes for Tamoxifen precursors and glycoside derivatives	Five-fold cross-validation on RadicalDB; baseline, reaction-subtype, invalid-SMILES, case-study, and attention analyses	Combined cross-validation with reaction-specific and interpretability analyses. Barton–McCombie reactions had low accuracy because the model struggled to identify the relevant C-H bond	Xu J. et al. (2025)
	GTA (Graph Truncated Attention; template-free seq2seq framework)	Datasets from USPTO-50k and USPTO-full	Achieved new state-of-the-art exact match Top-1 and Top-10 accuracies of 51.1% and 81.6% on USPTO-50k, and 46.0% and 70.0% on USPTO-full without reaction class information; surpassed prior graph-based template-free models by 2% (Top-1) and 7% (Top-10) on USPTO-50k, and by over 6% for both metrics on USPTO-full	Top-k benchmarking on USPTO-50K and USPTO-full; five-seed experiments and GTA-self/GTA-cross ablation studies	Demonstrated reproducibility, component effects, and scalability. Relies on approximated atom mapping from FMCS algorithm and soft loss guidance due to incomplete sequence mapping during decoding	Seo et al. (2021)
ADMET profiling and toxicity prediction	Evolved Graphormer (Transformer integrating fingerprints and physicochemical features via tree models)	Curated ChEMBL dataset covering logS, CL, Caco-2, PPB, and LD50 profiles	Achieved new state-of-the-art performance on ADME/Tox benchmarks; demonstrated an average SMAPE of 18.9 and a Pearson Correlation Coefficient (PCC) of 0.86; enhanced model interpretability by incorporating traditional molecular descriptors	Five-endpoint testing using *R* ^2^, SMAPE, and PCC; comparison with iDrug, ADMETLab, QikProp, and Discovery Studio	Evaluated multiple ADME/Tox properties against established tools. Comparator training data may overlap with the public test data; 3D structural information was not incorporated	Shao et al. (2024)
	Deep-PK (Utilizing D-MPNN/Directed Message Passing Neural Networks and graph-based signatures)	Multi-source dataset (ADMETlab 2.0, toxCSM, pkCSM, etc.) covering 73 endpoints	Yielded best predictive performance across all 73 endpoints (8 absorption, 5 distribution, 13 metabolism, 3 excretion, 35 toxicity). Provides tools for the characterization and optimization of small molecules	Random 8:1:1 splits, three-fold cross-validation, benchmark comparison, and chemical-space coverage analysis across 73 endpoints	Combined broad endpoint coverage, confidence reporting, and repeated validation. Accuracy decreases for structurally dissimilar compounds, four endpoints showed degradation, and optimization may not improve ADMET.	Myung et al. (2024)
	Multi-model GCN approach (Tailored GCN architectures for regression and binary classification; integrated with virtual screening)	TOXRIC toxicity datasets (acute, carcinogenicity, hERG, mutagenicity, hepatotoxicity)	Demonstrated high predictive reliability; achieved Pearson R of 0.76 (intraperitoneal), 0.74 (intravenous), and 0.65 (oral) for acute toxicity; attained AUCs of 0.88 for mutagenicity, 0.79 for hepatotoxicity, 0.77 for hERG cardiotoxicity, and 0.69 for carcinogenicity; successfully integrated these models into a virtual screening pipeline to identify low-toxicity candidates	Regression and classification testing across five toxicity types; approved-drug threshold calibration and virtual-screening evaluation	Used endpoint-specific models and an approved-drug dataset to define prediction thresholds. Small dataset size remains the main limiting factor, leading to performance variations across endpoints (carcinogenicity AUC = 0.69) and a lack of explicit toxicity explanations	Saravanan et al. (2024)
​	ADMETlab 3.0 (Utilizing a multi-task DMPNN architecture integrated with molecular descriptors)	Large-scale dataset (>400K molecules) covering 77 ADMET endpoints from ChEMBL and PubChem	Achieved superior performance in accuracy and robustness while maintaining high calculation speed; implemented uncertainty estimation to facilitate confident decision support; introduced API functionality for programmatic high-throughput integration into discovery pipelines	Random 8:1:1 splits for 77 models, five repeated training runs, model comparisons, runtime testing, and uncertainty evaluation	Used repeated validation, multiple metrics, broad endpoint coverage, and uncertainty estimates. Complex endpoints (e.g., melting point) were limited by data size; descriptor integration slightly reduced speed, and uncertainty estimates were restricted to the API.	Fu et al. (2024)
Drug repurposing	GraphConvMol (GCN-based model within the DeepChem framework using RDKit features)	DUD-E JAK2 dataset (107 actives, 6,500 decoys) screened against 3,105 FDA-approved drugs	Successfully identified four novel JAK2 inhibitors (Ribociclib, Topiroxostat, Amodiaquine, and Gefitinib). Results were validated through molecular docking and biological assays, demonstrating inhibitory activities comparable to the FDA-approved inhibitor Tofacitinib	Five-fold cross-validation on DUD-E; molecular docking against JAK2; JAK2 kinase assays using tofacitinib as reference	Kinase assays confirmed the DL and docking results. Limited chemical diversity in the FDA-approved and DUD-E datasets may restrict generalizability to other chemical spaces	Yasir et al. (2024)
	DeepDrug (Expert-guided GNN framework encoding a weighted, signed directed heterogeneous biomedical graph)	Multimodal data from DrugBank, DrugCentral, ChEMBL, BindingDB, and STRING	Identified a synergistic five-drug combination (Tofacitinib, Niraparib, Baricitinib, Empagliflozin, and Doxercalciferol) for Alzheimer’s disease; concurrently modulated neuroinflammation, mitochondrial dysfunction, and glucose metabolism; implemented high-order selection via diminishing return-based thresholds	Ten-fold cross-validation with nested validation and held-out testing; robustness and statistical analyses; comparison with biological, animal, epidemiological, and clinical evidence	Consistent cross-fold performance and prior evidence supported the selected candidates. Limited Alzheimer’s disease data, simplified graph representation, and model opacity remain concerns; the proposed combination awaits *in vitro* and *in vivo* testing	Li V. O. K. et al. (2025)
​	DLEVDA (Ensemble framework integrating CNN for feature extraction and XGBoost for virus–drug association classification)	455 drug–virus associations (219 drugs/34 viruses); NCBI genomes and DrugBank fingerprints	Achieved AUC of 0.8897 and prediction accuracy of 0.8571. Successfully prioritized 12 top-ranked anti-viral drugs (e.g., Ribavirin, Nitazoxanide), with 9 out of 12 candidates validated by literature evidence (PMID)	Repeated five-fold cross-validation, a held-out test set, method and classifier comparisons, evaluation on another dataset, and literature-supported case studies	Evaluation across classifiers, datasets, and literature supported predictive robustness. Randomly selected negatives may contain true associations, while experimentally confirmed drug–virus associations are limited	Deepthi et al. (2021)
	MVGAE (Integrated framework combining multi-view graph auto-encoders with network-informed adaptive learning)	Integrated networks from BioGRID, CTD, SynLethDB, and DGIdb	Predicted 7 driver genes and 9 synthetic lethality (SL) partners (e.g., ATR, RAD51) as NSCLC therapeutic targets; identified repurposed drugs including Pazopanib and Bortezomib; validated computational results against clinical biomarkers and established oncology literature	Five-fold stratified cross-validation and baseline comparisons; expression–sensitivity analysis, molecular docking, literature comparison, and ClinicalTrials.gov screening	Multiple computational and existing-evidence sources supported candidate prioritization. Docking and correlation analyses are not experimental confirmation; external cohorts and *in vitro*/*in vivo* validation remain necessary	Hu et al. (2026)

**FIGURE 1:**
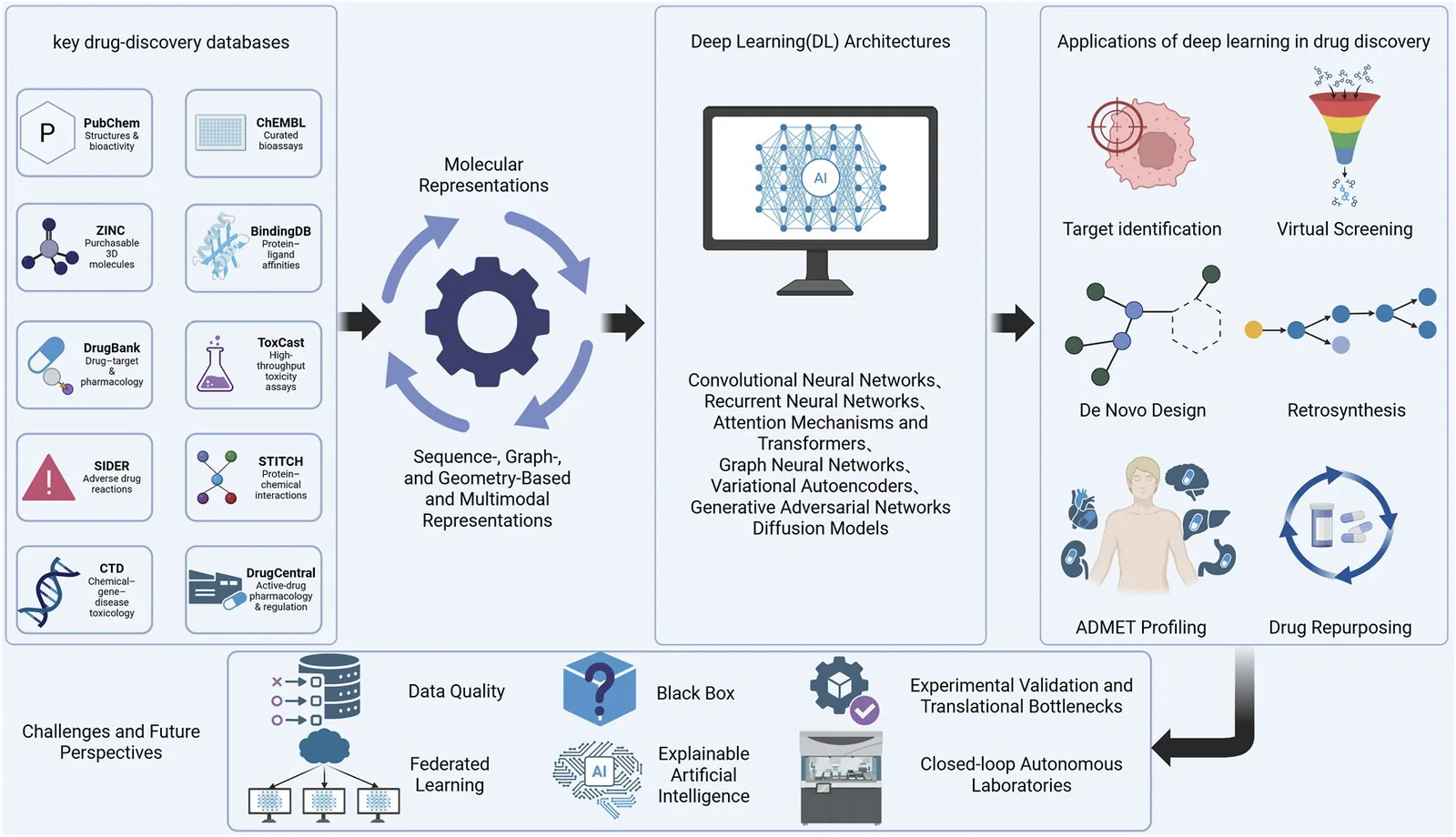
Conceptual overview of deep learning in drug discovery presented in this review. The figure outlines the progression from data foundations and molecular representations to DL architectures, major applications, and future directions.

This review aims to comprehensively and systematically summarize recent advancements in DL methodologies for drug discovery. First, we outline the critical data foundations and the principles of molecular representation learning underpinning artificial intelligence therapeutics. We then systematically examine the core DL architectures employed to process these complex biological and chemical datasets. Beyond providing a broad overview, this review offers a decision-oriented synthesis that links molecular representations to architecture-specific capabilities and evaluates model suitability and trade-offs across distinct drug-discovery scenarios. Subsequently, we discuss the diverse applications of DL across the discovery pipeline, including target identification, virtual screening, *de novo* molecular design, retrosynthetic analysis, ADMET profiling, and drug repurposing. Rather than interpreting reported performance in isolation, we further consider the heterogeneity of benchmarks, evaluation criteria, and validation evidence when assessing the reliability and practical relevance of different approaches. Finally, we critically evaluate the persistent challenges in translating computational predictions into clinical success, with a particular focus on data scarcity, model opacity, experimental validation bottlenecks, and prohibitive computational overhead. To address these limitations, we explore emerging solutions driven by federated learning, causality-aware Explainable AI (XAI), closed-loop autonomous laboratories, and efficiency-driven model optimization that will shape the next-generation of pharmaceutical research and development strategies. The scope and limitations of the present review are also acknowledged, together with priorities for future research aimed at improving methodological comparability, validation rigor, reproducibility, and translational relevance. Figure 1 summarizes the overall framework of this review.

## 2 Literature search and study selection

To improve transparency and reproducibility, a structured literature search was conducted in PubMed, Web of Science, and Scopus for English-language publications from January 2016 to July 2026. The search combined (“deep learning” OR “artificial intelligence” OR “neural network”) with (“drug discovery” OR “target identification” OR “virtual screening” OR “*de novo* molecular design” OR “retrosynthesis” OR “ADMET” OR “drug repurposing”). Reference lists of relevant publications were additionally screened, and earlier seminal studies were retained when necessary to describe foundational datasets, representations, or architectures. After duplicate removal, titles and abstracts were screened, followed by full-text assessment. Studies were included when DL constituted a substantive methodological component of pharmaceutical discovery and sufficient information was reported on the model, data, application, or evaluation; publications lacking direct methodological relevance were excluded. For each selected study, information on molecular representation, architecture, application, dataset, evaluation metrics, principal findings, validation strategy, and validation strengths and limitations was extracted and qualitatively synthesized, as summarized in Tables 1, 2.

**TABLE 2:** Comparative summary, computational/data profiles, and scenario-based suitability of DL architectures in drug discovery.

DL architectures	Primary data representation	Core mechanism and strengths	Key drug discovery applications	Key limitations and bottlenecks	Computational and data profile	Scenario-based suitability
CNNs	1D strings, 2D images, 3D voxel grids	Extracts local, translation-invariant features via sliding filters, excelling at learning functional group motifs	Toxicity prediction, binding site localization, RNA-small molecule affinity	Localized focus overlooks global sequence context; grid-based inputs obscure atomic precision and lack 3D equivariance, failing to capture non-Euclidean molecular complexity	High parallelism; near-linear scaling, but 3D inputs are memory-intensive. Moderate data needs; transfer learning supports small datasets	Local feature extraction, toxicity prediction, molecular images, spectra and voxelized binding-pocket analysis
RNNs	1D sequences (SMILES, SELFIES)	Maintains internal hidden states to capture sequential grammar and syntactical dependencies	Early sequence-based affinity prediction, *de novo* molecular generation	Fundamental disconnect from 3D reality and topological features; prone to information decay in long sequences; lacks native spatial awareness	Low per-step cost but serial execution; slow for long sequences. Moderate-to-low data efficiency	SMILES generation and sequential molecular modeling when sequence length and computational resources are limited
Attention mechanisms and transformers	1D sequences, hybrid/multimodal	Utilizes self-attention to weigh pairwise token relationships simultaneously, excelling at long-range dependencies	Foundation chemical language modeling, bidirectional feature interaction, lead optimization	Quadratic scaling complexity and memory burden. Highly data-hungry and prone to overfitting. Attention maps may lack biological causality	Parallel training but quadratic attention cost. Data-intensive from scratch; strong few-shot transfer after pre-training	Chemical language modeling, long-range dependency learning, multimodal integration and protein–ligand sequence analysis
GNNs	2D molecular graphs, 3D spatial graphs	Ingests non-Euclidean connectivity via message-passing, explicitly modeling topology without ordering biases	Drug-target affinity (DTA), ADMET profiling, target identificationetc.	Susceptible to over-smoothing in deep layers. Standard models lack explicit 3D spatial awareness regarding bond angles and conformations	Cost scales with nodes, edges and layers; efficient for sparse graphs. Good data efficiency from structural inductive bias	Molecular property prediction, ADMET profiling, DTI prediction and topology-based interaction modeling
VAEs	Diverse (mapped to latent space)	Compresses structural features into a continuous probabilistic latent space for smooth interpolation	Multi-parameter optimization (MPO), target-aware *de novo* design	Risk of posterior collapse and limited structural diversity. Inherent tension between reconstruction fidelity and latent space regularity	Stable training and fast one-pass generation. Moderate data needs; conditional adaptation requires labeled properties	Latent-space exploration, scaffold interpolation, multi-property optimization and rapid candidate generation
GANs	Diverse (generative outputs)	Employs a competitive learning paradigm to iteratively refine and synthesize highly realistic compounds	High-throughput chemical space exploration, target-specific lead synthesis	Training instability and mode collapse. Gradients are incompatible with discrete data, while reinforcement learning risks reward-hacking	Costly adversarial training but fast one-pass inference. Data-sensitive; weak few-shot stability	Rapid generation of realistic, distribution-matched molecular libraries when training stability can be controlled
Diffusion models	3D Cartesian coordinates, multi-modal	Iteratively refines molecular structures from Gaussian noise through reverse denoising for high spatial fidelity	3D pocket-conditioned design, spatial conformation generation, fragment linking	Substantial computational overhead from iterative sampling. Difficulty in ensuring physical realism such as bond length and connectivity	High sampling cost due to iterative denoising. Requires high-quality structural data; transfer learning may aid adaptation	Pocket-conditioned 3D generation, conformational design, fragment linking and structure-based campaigns prioritizing geometric fidelity

## 3 The data foundation of AI-driven drug discovery

The efficacy of AI in drug discovery is intrinsically tethered to the scale and diversity of public biomedical repositories. Massive libraries like PubChem (Kim et al., 2023), ChEMBL (Zdrazil, 2025), and ZINC (Tingle et al., 2023) define the explorable chemical space, providing structural and bioactivity data crucial for virtual screening and *de novo* generation. Advanced applications, including drug-target interaction (DTI) prediction and drug repurposing, rely on specialized relational datasets like BindingDB (Liu T. et al., 2025) and DrugBank (Knox et al., 2024). Furthermore, evaluating safety profiles and systemic interactions to mitigate clinical attrition depends on resources like Toxcast (Richard et al., 2016), SIDER (Kuhn et al., 2016), STITCH (Szklarczyk et al., 2016), CTD (Davis et al., 2021), and DrugCentral (Avram et al., 2023), all of which are summarized in Table 3. Collectively, these repositories constitute an indispensable discovery ecosystem.

**TABLE 3:** Key datasets in the field of drug discovery.

Dataset	Description	Link	APIs	References
PubChem	Aggregates structural and bioactivity data across millions of compounds, providing foundational features for computational drug discovery	https://pubchem.ncbi.nlm.nih.gov	Yes	Kim et al. (2023)
ChEMBL	Curates large-scale bioassay data linking chemical substances to biological activities, essential for virtual screening and lead optimization	https://www.ebi.ac.uk/chembl/	Yes	Zdrazil (2025)
ZINC	A multi-billion-scale database of commercially available 3D molecules, designed to facilitate ultra-large-scale virtual screening and ligand discovery	https://cartblanche22.docking.org/	Yes	Tingle et al. (2023)
BindingDB	Compiles experimentally measured protein-ligand binding affinities, providing critical quantitative ground truth for training affinity prediction models	https://www.bindingdb.org	Yes	Liu T. et al. (2025)
DrugBank	A gold-standard resource integrating detailed drug, target, and pharmacological data, widely utilized for computational drug repurposing	https://go.drugbank.com	Yes	Knox et al. (2024)
Toxcast	Provides high-throughput *in vitro* assay data evaluating chemical toxicity, serving as a benchmark for ADMET prediction models	https://www.epa.gov/comptox-tools/exploring-toxcast-data	Yes	Richard et al. (2016)
SIDER	Catalogs marketed drugs and their adverse drug reactions (ADRs), facilitating computational modeling of safety profiles and off-target effects	http://sideeffects.embl.de	Yes	Kuhn et al. (2016)
STITCH	Integrates protein-chemical interaction networks from diverse sources to facilitate drug target identification	http://stitch.embl.de	Yes	Szklarczyk et al. (2016)
CTD	Correlates toxicological information across chemicals, genes, and diseases, offering harmonized cross-species data to inform target-directed therapies	http://ctdbase.org/	Yes	Davis et al. (2021)
DrugCentral	Compiles structural, biological, and regulatory data for active drugs, supporting comprehensive pharmacological profiling and digital repositioning efforts	http://drugcentral.org	Yes	Avram et al. (2023)

Abbreviations: APIs, application programming interfaces; BindingDB, binding database; ChEMBL, chemical database of the european molecular biology laboratory; CTD, comparative toxicogenomics database; SIDER, side effect resource; STITCH, search tool for interacting chemicals; Toxcast, Toxicity Forecaster; ZINC, ZINC, Is Not Commercial. In this table, each entry includes the abbreviated database name, concise description, web link, API, availability, and representative references.

## 4 Methodological foundations: molecular representations and deep learning architectures

The fundamental challenge in applying artificial intelligence to pharmaceutical sciences lies in bridging the gap between physical chemical reality and computational abstraction. For neural networks to effectively learn structure-activity relationships (SAR) and predict complex biological interactions, raw molecular entities must be translated into machine-readable formats. This section systematically reviews the principal paradigms of molecular representation learning and the core DL architectures driving drug discovery.

### 4.1 Molecular representation learning in drug discovery

#### 4.1.1 Sequence-based representations

One-dimensional representations encode molecular topologies as linear text strings, facilitating the direct application of Natural Language Processing (NLP) models to chemical data. The Simplified Molecular Input Line Entry System (SMILES) is the most widely adopted standard in this category, representing atoms, bonds, and ring structures through specific alphanumeric characters. While highly efficient for pre-training large chemical language models, SMILES strings possess inherent limitations regarding structural robustness. Specifically, generative DL models often produce syntactically incorrect and chemically invalid SMILES sequences during *de novo* design (Grisoni, 2023). To resolve this fragility, alternative text representations like Self-Referencing Embedded Strings (SELFIES) have been developed. SELFIES ensures complete chemical validity for every generated string by design, thereby significantly enhancing the efficiency and reliability of generative discovery pipelines (Krenn et al., 2020).

#### 4.1.2 Graph-based representations

Despite the computational convenience of linear strings, molecules are intrinsically structural entities. Graph-based representations address this reality by modeling compounds as non-Euclidean mathematical graphs, where atoms function as nodes and chemical bonds act as connecting edges. This approach naturally encapsulates the topological connectivity and local chemical environments of a molecule. By accurately mapping essential pharmacophores and aromatic rings without the sequence ordering biases inherent in 1D strings, 2D molecular graphs have become the dominant and most intuitive format for deploying Graph Neural Networks (GNNs) (Liu S. et al., 2025; Koge et al., 2026). This structural fidelity makes graph representations exceptionally well-suited for high-throughput virtual screening and accurate molecular property prediction (Rasool et al., 2025).

#### 4.1.3 Geometry-based representations

While 2D graphs effectively map chemical connectivity, they fundamentally lack spatial awareness. Biological interactions, particularly those dictating drug-target binding affinities, are strictly governed by spatial conformations, stereochemistry, and dynamic electrostatic surfaces. Geometry-based representations model molecules using 3D Cartesian coordinates, point clouds, or voxel grids to provide this critical biophysical context. These high-dimensional formats enable computational models to evaluate steric hindrance and binding pocket complementarity with unprecedented precision (Yu et al., 2023). The adoption of 3D representations is highly critical for advancing structure-based drug design and forms the foundational input for sophisticated 3D-equivariant neural networks (Pelaez et al., 2024).

Recent geometric deep learning approaches address this representational gap by enforcing equivariance to Euclidean transformations. In E(3)- or SE(3)-equivariant networks, rotating or translating the input molecular coordinates induces a corresponding transformation of vector- or tensor-valued features, while scalar predictions such as binding affinity remain invariant. This symmetry-aware inductive bias reduces the need for rotational data augmentation and enables direct learning from atomic coordinates, interatomic distances, orientations, and higher-order angular relationships. Recent models illustrate the resulting breadth of application: the Generalist Equivariant Transformer represents heterogeneous 3D complexes as geometric graphs of sets and learns interaction patterns across proteins, small molecules, and nucleic acids (Kong et al., 2023), whereas DiffBindFR applies an SE(3)-equivariant diffusion framework to jointly model ligand motion, ligand flexibility, and binding-pocket side-chain torsions for flexible protein-ligand docking (Zhu et al., 2024). These developments allow geometric representations to bridge 2D connectivity with conformational and interaction-level information that is directly relevant to structure-based drug discovery.

Equivariance alone, however, does not guarantee physically realistic molecular behavior. Current frameworks increasingly incorporate physical priors through force-field terms, potential-energy surfaces, conformer energies, and geometry-dependent interaction constraints. For example, FENNIX combines a local equivariant neural network with physically motivated electrostatic and dispersion terms, using predicted atom-in-molecule properties to parameterize long-range interactions (Plé et al., 2023). More recently, EquiCPI integrates SE(3)-equivariant message passing with physics-guided conformer reranking to improve structure-aware compound-protein interaction prediction (Nguyen and Kang, 2025). Such hybrid strategies complement data-driven geometric features with energetic plausibility, thereby improving transferability to unseen conformations and reducing the risk of learning geometrically valid but energetically unfavorable structures. Nevertheless, their performance remains sensitive to conformer quality, structural uncertainty, and the completeness of the underlying physical approximations, while atomistic processing and higher-order equivariant operations impose substantial computational and memory costs.

#### 4.1.4 Hybrid and multimodal representations

Recognizing that no single format captures the entirety of a molecule’s physicochemical profile, researchers are increasingly adopting hybrid and multimodal representations (Lu et al., 2024). These advanced frameworks simultaneously process multiple data streams, combining the rapid sequence processing of 1D strings with the topological accuracy of 2D graphs and the spatial fidelity of 3D coordinates. Furthermore, cutting-edge foundation models now integrate unstructured textual descriptions, biomedical knowledge graphs, and multi-omics profiles alongside chemical structures (Zang et al., 2026). By aligning these diverse modalities into a unified latent space through contrastive learning, hybrid representations empower DL models to achieve superior generalization and perform highly contextualized tasks in modern therapeutics (Zhang T. et al., 2024).

### 4.2 Deep learning architectures in drug discovery

#### 4.2.1 Convolutional neural networks

**FIGURE 2:**
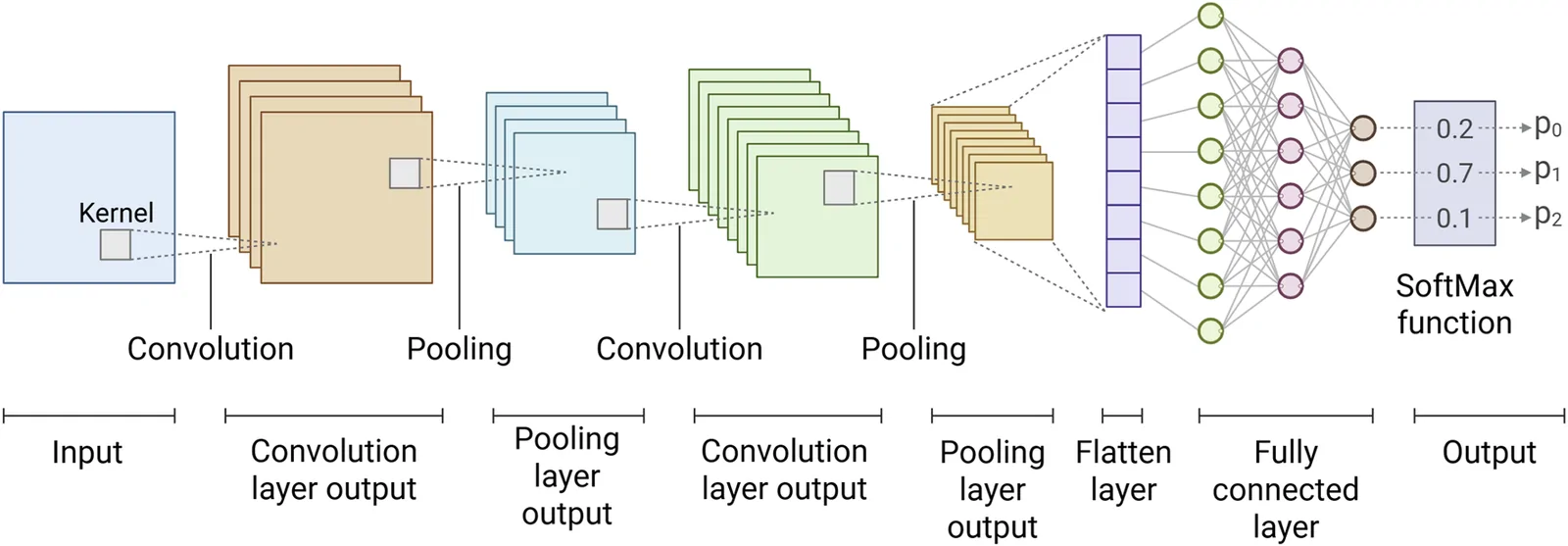
Standard CNN architecture for molecular informatics. The network hierarchically extracts translation-invariant features from molecular inputs (e.g., SMILES matrices or voxel grids) via alternating convolution and pooling layers. Flattened feature maps are processed through fully connected layers and a SoftMax function for downstream analysis.

Convolutional Neural Networks (CNNs) served as foundational pillars for early artificial intelligence applications in drug design. CNNs are highly proficient at extracting local, translation-invariant patterns through the application of sliding convolutional filters (Figure 2). In chemoinformatics, 1D-CNNs are frequently deployed to process SMILES strings, while 2D-CNNs and 3D-CNNs are utilized to evaluate spatial molecular representations such as images and voxel grids (Wang Y. et al., 2022). By autonomously learning functional group motifs, CNNs provide a robust baseline for toxicity and property prediction.

Recent studies exemplify the versatility of these convolutional architectures when augmented with advanced learning paradigms. For instance, Zhao et al. developed AttentionDTA (Zhao et al., 2023), a model utilizing dual 1D-CNNs to independently extract semantic features from drug SMILES strings and protein amino acid sequences. By integrating a two-side multi-head attention mechanism, the framework not only achieves state-of-the-art drug–target affinity (DTA) prediction but also provides mechanistic interpretability by effectively localizing binding sites. Expanding CNNs into multimodal ecosystems, Park and Lee introduced MoltiTox (Park and Lee, 2025), which employs 1D-CNNs and 2D-CNNs to process 13C NMR spectra and 2D molecular images, respectively, alongside GNNs and Transformers. Fused via an attention mechanism, this heterogeneous approach captures complementary chemical insights, significantly enhancing the robustness of molecular toxicity prediction across the Tox21 benchmark. Furthermore, to navigate complex spatial environments, Sun and Gao proposed Rlaffinity (Sun and Gao, 2024), a pioneering model for predicting RNA–small molecule binding affinity. By coupling a 3D-CNN with contrastive self-supervised pre-training, the architecture accurately extracts both global pocket topologies and local nucleotide interactions from 3D structures, underscoring the profound capability of 3D-CNNs in advancing RNA-targeted virtual screening.

Despite their strengths, CNNs are constrained by localized feature extraction, which often overlooks global context in 1D sequences. In higher dimensions, grid-based inputs obscure atomic precision and lack natural 3D equivariance, necessitating extensive data augmentation for protein-ligand modeling. These limitations ultimately prevent CNNs from fully capturing the complex, non-Euclidean connectivity inherent in molecular entities.

#### 4.2.2 Recurrent neural networks

**FIGURE 3:**
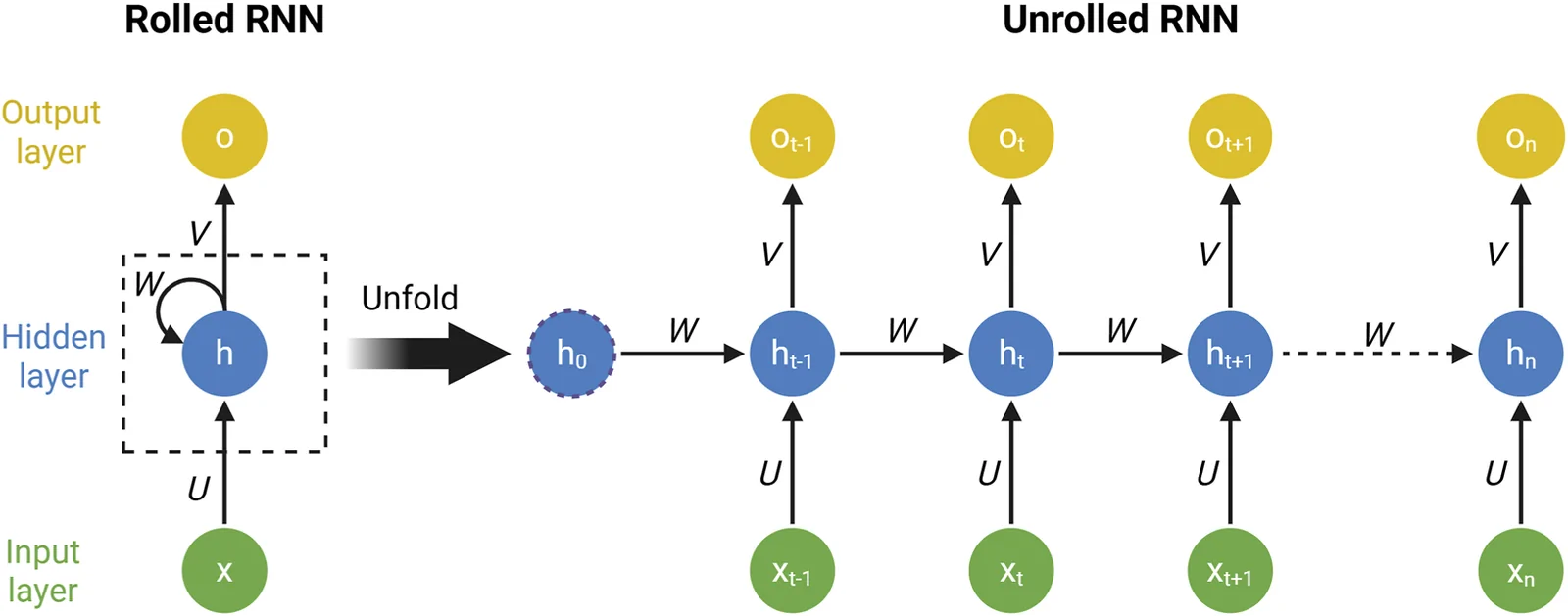
Structural overview of a Recurrent Neural Network (RNN). The left panel illustrates the compressed (rolled) representation with an internal hidden state (h) that loops over time. The right panel depicts the unrolled network across sequential time steps (t−1, t, t+1), demonstrating how the hidden state propagates sequential dependencies from past inputs (x) to inform future outputs (o).

Parallel to the development of spatial feature extractors, Recurrent Neural Networks (RNNs) and their advanced variants, including Long Short-Term Memory (LSTM) networks and Gated Recurrent Units (GRUs), were specifically designed to handle sequential chemical data. By maintaining internal hidden states, RNNs effectively capture the sequential grammar and syntactical dependencies inherent in one-dimensional molecular representations like SMILES strings (Figure 3). This capability made RNNs the initial architecture of choice for early *de novo* molecular generation and sequence-based binding affinity predictions (Tong et al., 2021). However, the sequential processing nature of RNNs inherently limits computational parallelization and presents significant challenges in maintaining context when dealing with exceptionally long molecular sequences. These fundamental architectural constraints ultimately catalyzed the field’s transition toward attention-based models capable of global sequence comprehension.

Despite these inherent limitations, recurrent architectures continue to demonstrate robust utility when strategically hybridized with contemporary learning paradigms or expanded into bidirectional frameworks. For instance, in *de novo* molecular generation, Gou et al. developed CNSMolGen (Gou et al., 2024), employing a Bidirectional RNN (Bi-RNN) to specifically design Central Nervous System (CNS) therapeutics. By coupling the generative model with transfer learning, they successfully produced novel, synthesizable candidates optimized for the serotonin transporter (SERT), subsequently validated via physics-based induced-fit docking. In the context of drug-target affinity (DTA) prediction, Qiu et al. proposed LSTM-SAGDTA (Qiu et al., 2024), an architecture that synergizes an LSTM module for processing sequential protein characterizations (SeqVec) with self-attentive graph pooling for molecular structures, yielding high-precision predictions with superior computational efficiency. Furthermore, addressing molecular toxicity, Liu et al. introduced the MTBG framework (Liu J. et al., 2023), which utilizes a Bidirectional GRU (BiGRU) to extract deep syntactical dependencies from SMILES strings in tandem with GraphSAGE for topological graph representation. Evaluated on the Tox21 benchmark, this dual-pathway model achieved robust predictive performance, illustrating how advanced RNN variants continue to serve as critical sequential processing engines within modern, multimodal pipelines.

In conclusion, the reliance of RNNs on linear SMILES strings creates a fundamental disconnect from the 3D reality of molecular structures. This sequence-based approach often fails to capture essential topological features, which can lead to the design of chemically unstable or synthetically complex candidates. Additionally, information decay remains a persistent challenge when processing exceptionally long sequences. Because these models lack native spatial awareness, they are increasingly relegated to auxiliary roles within advanced architectures that directly process graph-based or 3D data.

#### 4.2.3 Attention mechanisms and transformers

**FIGURE 4:**
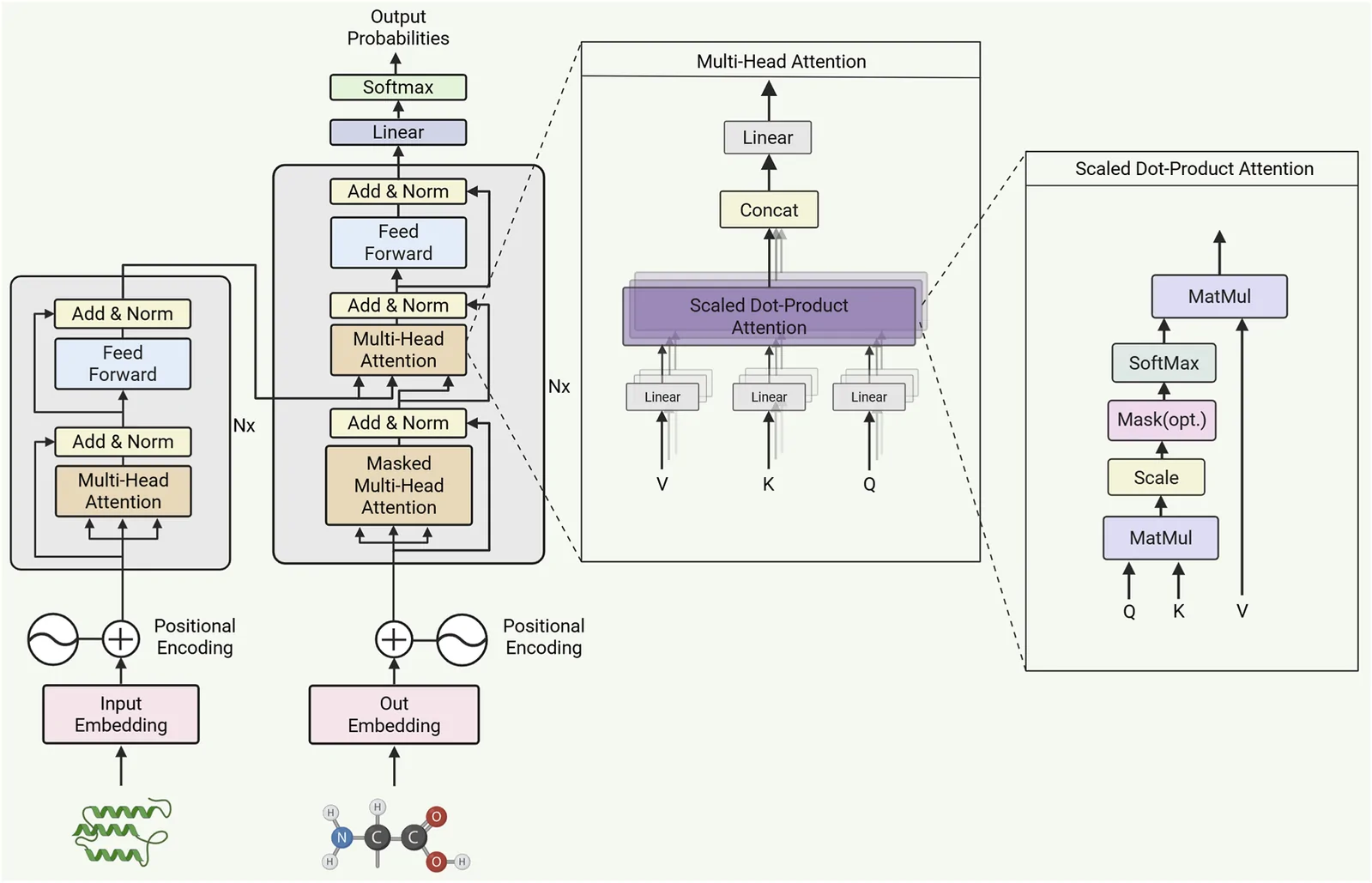
Architecture of the transformer model. The schematic details the integration of positional encoding with initial embeddings. It highlights the core stacked encoder and decoder modules, which heavily rely on multi-head self-attention mechanisms and feed-forward networks, enabling the model to simultaneously weigh global contextual relationships and capture long-range chemical dependencies.

To overcome the inherent limitations of recurrent architectures, the Transformer model has emerged as a dominant paradigm in molecular sequence modeling. The core innovation of this architecture is the self-attention mechanism, which allows the model to dynamically evaluate and weigh the pairwise relationships between all input tokens simultaneously, regardless of their absolute distance in the sequence (Figure 4). This non-sequential processing capability is exceptionally well-suited for capturing long-range chemical dependencies, such as interactions between distal functional groups within a large macro-molecule. Building upon this architecture, Chemical Language Models (CLMs) have revolutionized property prediction. By pre-training on massive unannotated chemical databases containing tens of millions of molecules, foundation models such as ChemBERTa and MoLFormer acquire a profound underlying understanding of chemical syntax and semantics (Ross et al., 2022; Singh et al., 2026). While ChemBERTa employs a standard BERT-based framework pre-trained on up to 100 million compounds, MoLFormer leverages linear attention and rotary embeddings to scale pre-training to 1.1 billion molecules for enhanced structural awareness. Such pre-training facilitates seamless fine-tuning for downstream tasks, ensuring robust predictive accuracy within the data-scarce environments of specialized biological assays. More recently, molecular LLMs have extended this sequence-based paradigm by jointly processing chemical strings, natural-language instructions, protein-related information and other biological contexts. Rather than learning chemical syntax in isolation, these models seek to connect molecular representations with biological semantics, thereby supporting *de novo* hit generation, molecular property prediction and multi-objective lead optimization. Structure-aware tokenization and multimodal fusion can further incorporate molecular topology and three-dimensional geometric constraints, partially overcoming the limited spatial expressiveness of purely one-dimensional chemical representations (Chen et al., 2026).

Expanding on these core principles, recent research has tailored Transformer architectures to refine drug-target interaction (DTI) prediction and molecular design. In virtual screening, sequence-based models like Ligand-Transformer utilize protein amino acid sequences and ligand topologies to predict binding affinities and the characterization of population shifts in free energy landscapes (Zhang S. et al., 2025). To address the complexities of multi-modal data, frameworks such as CAT-DTI and CoaDTI have introduced sophisticated attention mechanisms for bidirectional feature interaction (Huang et al., 2022; Zeng et al., 2024). Specifically, CAT-DTI integrates CNN-Transformer blocks for protein distance modeling with a GCN for drug features, utilizing a conditional domain adversarial network (CDAN) to ensure robust generalization across out-of-distribution data. In contrast, CoaDTI employs a co-attention mechanism to fuse GraphSAGE-derived molecule features with Transformer-encoded protein representations, leveraging transfer learning from pre-trained language models to mitigate data scarcity and provide mechanistic interpretability through attention visualization. These advancements in DTI prediction are complemented by specialized screening and optimization frameworks. For instance, the HBCVTr model adopts a double-encoder Transformer hybrid to achieve high-throughput screening against viral targets, successfully identifying novel inhibitors for HBV and HCV (Meewan et al., 2024). Beyond identification, Transformers facilitate precise lead optimization. Generative models like TransAntivirus utilize IUPAC nomenclature—which aligns closely with natural language—to perform functional-group-level edits for antiviral analogue design (Mao et al., 2024). Furthermore, by integrating multi-property constraints, recent architectures combining Graph-BERT with Constraints-Transformers allow for the simultaneous optimization of pharmacokinetic (ADMET) profiles and biological activity (Yang et al., 2023), ensuring that enhanced lead compounds retain their target selectivity while strictly preserving the core molecular scaffold.

However, the implementation of Transformers also presents significant challenges. The quadratic scaling complexity of the self-attention mechanism imposes a heavy computational and memory burden, particularly when modeling exceptionally long protein sequences. These models are also notably data-hungry, requiring massive pre-training datasets to achieve true generalization and avoid overfitting on small, biased experimental assays. Furthermore, while attention maps offer a degree of transparency, they do not always correlate with actual biological causality, which can occasionally lead to misleading interpretations of molecular interactions. Consequently, balancing model complexity with data availability remains a critical bottleneck for their widespread adoption in specialized therapeutic areas.

#### 4.2.4 Graph neural networks

**FIGURE 5:**
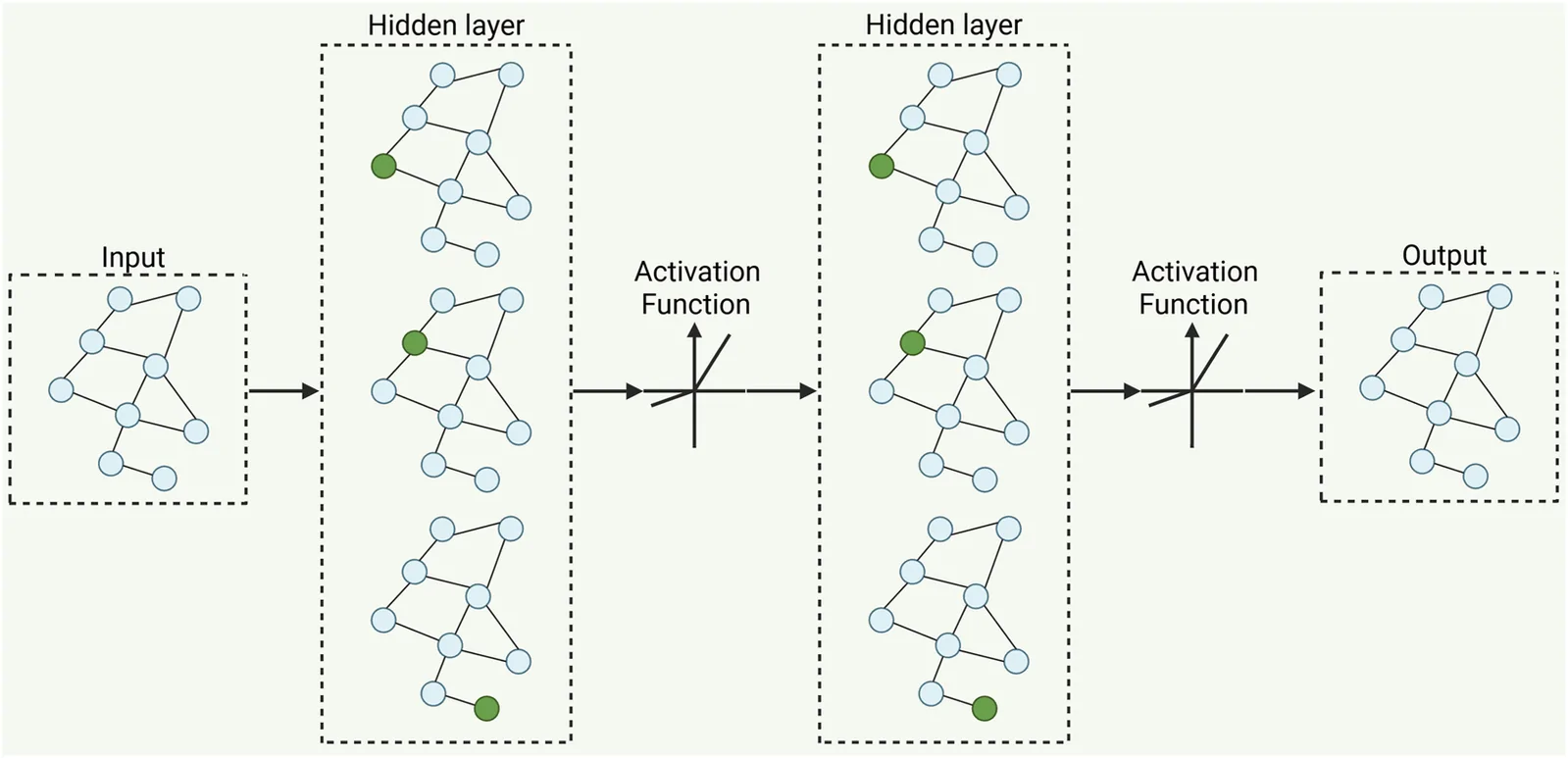
Illustration of a graph neural network (GNN). The architecture processes a molecular graph by executing an iterative message-passing protocol. In each hidden layer, node representations (e.g., the highlighted green atom) are recursively updated by aggregating feature information from local neighborhoods, followed by a non-linear activation function. These learned embeddings capture the complex structural dependencies to generate the final output for molecular-level or node-level tasks.

Given that molecules are fundamentally structured as topological entities, Graph Neural Networks (GNNs) have rapidly become the premier architecture for processing 2D molecular graphs (Figure 5). Diverging from grid-based CNNs or sequence-based Transformers, GNNs directly ingest the non-Euclidean connectivity of atoms and bonds. The inductive power of this framework derives from an iterative message-passing protocol. In implementations such as Graph Convolutional Networks (GCNs) and Message Passing Neural Networks (MPNNs), topological dependencies are encoded by recursively aggregating feature vectors from adjacent nodes (Kong et al., 2022; Wang Z. et al., 2022). This mechanism contextualizes each atom within its local chemical environment, accurately capturing the influence of specific pharmacophores and ring structures. Furthermore, Graph Attention Networks (GATs) introduce learnable attention coefficients to assign non-uniform importance to neighboring atoms, acting as a structural filter that dampens stochastic noise while amplifying critical functional signals (Ahmad et al., 2023). By explicitly modeling molecular topology without the strict ordering biases of SMILES strings, GNNs consistently achieve state-of-the-art performance in highly complex tasks, including drug-target interaction prediction and ADMET profiling (Cai et al., 2022; Wang et al., 2025).

Recent literature extensively validates these capabilities through specialized GNN architectures across diverse biomedical domains. In drug-target affinity (DTA) prediction, Liao et al. developed GSAML-DTA (Liao et al., 2022), which integrates self-attention mechanisms with GNNs and mutual information filtering to outperform baseline models while providing atomic-level interpretability of binding residues. Similarly, Li et al. proposed the Multiphysical GNN (MP-GNN) (Li et al., 2022), which innovates by mapping multiscale atomic interactions into element-specific graphs, thereby circumventing complex feature generation and achieving superior predictive performance across PDBbind datasets (v2007, v2013, v2016). This model also demonstrated high accuracy in evaluating 185 SARS-CoV/SARS-CoV-2 inhibitor complexes. For pharmacokinetic evaluation, Ito et al. employed multitask GNNs coupled with fine-tuning to effectively address data scarcity (Ito et al., 2025), achieving peak performance for 7 out of 10 ADME parameters while utilizing integrated gradients to provide chemical structure explainability during lead optimization. Beyond direct molecular profiling, GNNs excel in systems biology and network pharmacology. For instance, Wu et al. introduced deepCDG (Wu et al., 2025), a model utilizing shared-parameter GCN encoders and attention layers for multi-omics integration, successfully identifying cancer driver genes and complex gene modules with robust computational efficiency. Furthermore, Cui et al. developed the GraphSAGE-based GraphRepur to integrate drug-exposure expression profiles with topological drug-drug links (Cui et al., 2021), successfully predicting high-ranked repurposable therapeutic candidates for breast cancer that align with recent clinical reports.

Nevertheless, GNNs face inherent challenges such as over-smoothing, where repeated message-passing causes node representations to become indistinguishable in deep architectures. Many standard models also lack explicit 3D spatial awareness, failing to account for critical bond angles or conformational flexibility. These constraints can limit their effectiveness in tasks where precise geometric orientation is paramount, necessitating the development of more complex, equivariant graph frameworks.

#### 4.2.5 Variational autoencoders

**FIGURE 6:**
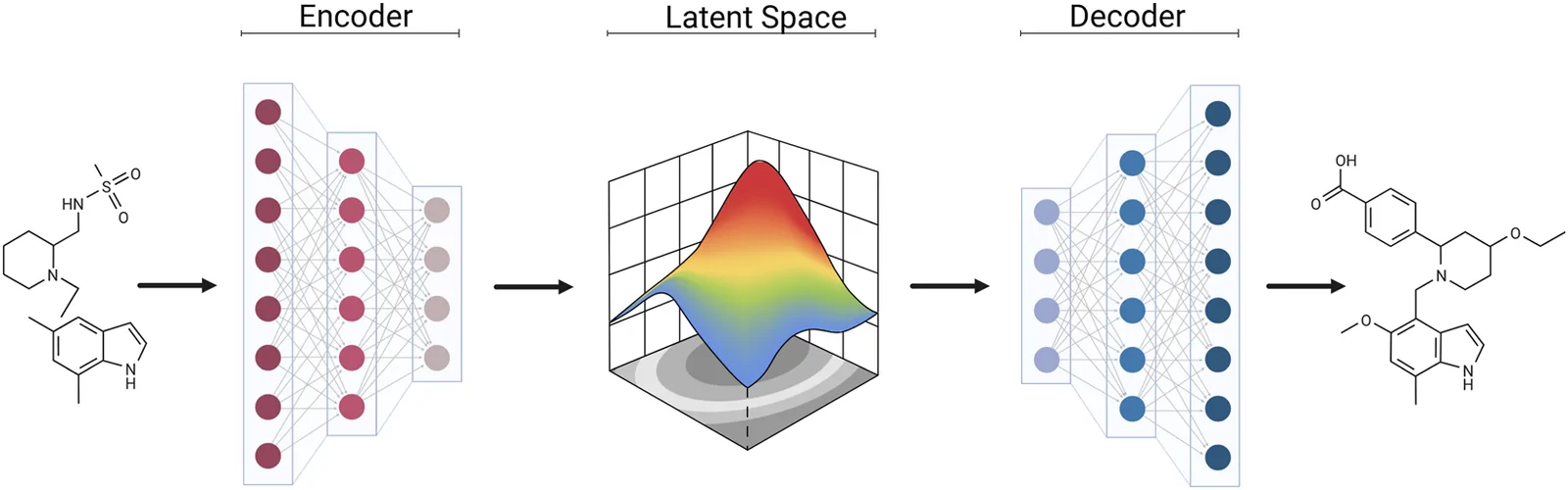
Schematic representation of a variational autoencoder (VAE) architecture. The model utilizes an encoder block to map discrete, structured molecular data into a continuous, probabilistic latent space. The decoder block subsequently reconstructs novel molecular graphs by sampling from this continuous distribution, facilitating smooth interpolation for property optimization.

Variational Autoencoders (VAEs) have fundamentally advanced *de novo* molecular design by mapping discrete chemical representations into a continuous, probabilistic latent space (Bhadwal et al., 2025). Recent methodological reviews classify VAEs alongside GANs, RNNs, Transformers, diffusion models, normalizing flows, and hybrid architectures, highlighting their distinct trade-offs in latent-space continuity, training stability, sampling efficiency, structural fidelity, and controllability (Chen and Xue, 2026). Within this broader taxonomy, VAEs are particularly valued for stable optimization and continuous representation learning in both distribution-learning and goal-directed molecular generation tasks. VAEs utilize an encoder-decoder architecture that compresses molecular features into a statistical distribution, allowing for the generation of structurally diverse compounds through latent space sampling (Figure 6). This continuous representation is particularly advantageous for multi-parameter optimization, as it enables smooth interpolation between known active molecules and facilitates gradient-based searches. By leveraging architectures such as variational autoencoders, researchers can effectively generate novel chemical structures tailored to specific target pockets (Tan et al., 2025).

Contemporary studies increasingly underscore the profound versatility of Variational Autoencoders (VAEs) in addressing intricate challenges within *de novo* drug design, with a particular focus on multi-objective optimization and generative diversity. For instance, Ngo et al. proposed TargetVAE (Khang Ngo and Son Hy, 2024), a target-aware model guided by a Protein Multimodal Network (PMN). By unifying 1D sequential, 2D topological, and 3D geometrical protein representations, TargetVAE successfully generates high-affinity, synthesizable ligands for arbitrary targets without requiring predefined pocket structures, demonstrating highly competitive binding affinity predictions on the PDBBind v2020 dataset. To tackle multi-objective property optimization, Dong et al. developed ScafVAE (Dong et al., 2025), a scaffold-aware architecture that expands accessible chemical space through perplexity-inspired fragmentation. This model effectively generated dual-target candidates against cancer resistance mechanisms, with molecular dynamics simulations confirming stable target interactions. Similarly, Lee and Min introduced the Molecular Graph Conditional VAE (MGCVAE) for multi-objective inverse design (Lee and Min, 2022). By conditionally guiding the latent space, MGCVAE successfully optimized dual physical properties (logP and molar refractivity) in 25.89% of generated molecules, representing a substantial improvement over the 0.66% yield achieved by standard MGVAEs. Furthermore, to address the pervasive issue of posterior collapse that limits generative diversity, Bhadwal et al. engineered PCF-VAE (Bhadwal et al., 2025). Evaluated on the MOSES benchmark, PCF-VAE effectively mitigated structural redundancy to achieve 100% molecular uniqueness, alongside exceptional validity (98.01%) and novelty (up to 95.01%), thereby ensuring robust and diverse exploration of the chemical space. In essence, this evolution shifts discovery from heuristic searches toward purposeful molecular engineering. A recent therapeutic-area-specific extension is CNSGT, which combines a variational autoencoder with self-attention and uses large-scale pretraining followed by transfer learning for dopamine transporter inhibitor generation. This hybrid design directly addresses the restrictive physicochemical requirements imposed by the blood-brain barrier: the generated candidates were chemically valid, achieved CNS multiparameter optimization scores above four and synthetic accessibility scores below 3, and included docked compounds with Glide scores below −8 kcal/mol. Molecular dynamics simulations further indicated stable binding conformations, while medicinal-chemist analysis supported the theoretical feasibility of the proposed synthetic routes (Chen et al., 2025b).

These methodological advancements highlight a continuous effort to overcome the intrinsic bottlenecks of the VAE architecture, most notably the persistent risk of posterior collapse that can stifle chemical diversity. Beyond generative variety, a fundamental tension remains between reconstruction fidelity and latent space regularity, often forcing a trade-off between molecular validity and structural novelty. Moreover, the probabilistic framework of VAEs, while more stable than adversarial training, may compromise the fine-grained geometric precision necessary for high-affinity binding predictions. Consequently, the practical utility of VAEs remains dependent on the integration of post-generation refinement protocols to ensure that candidates are both chemically feasible and biologically active.

#### 4.2.6 Generative adversarial networks

**FIGURE 7:**
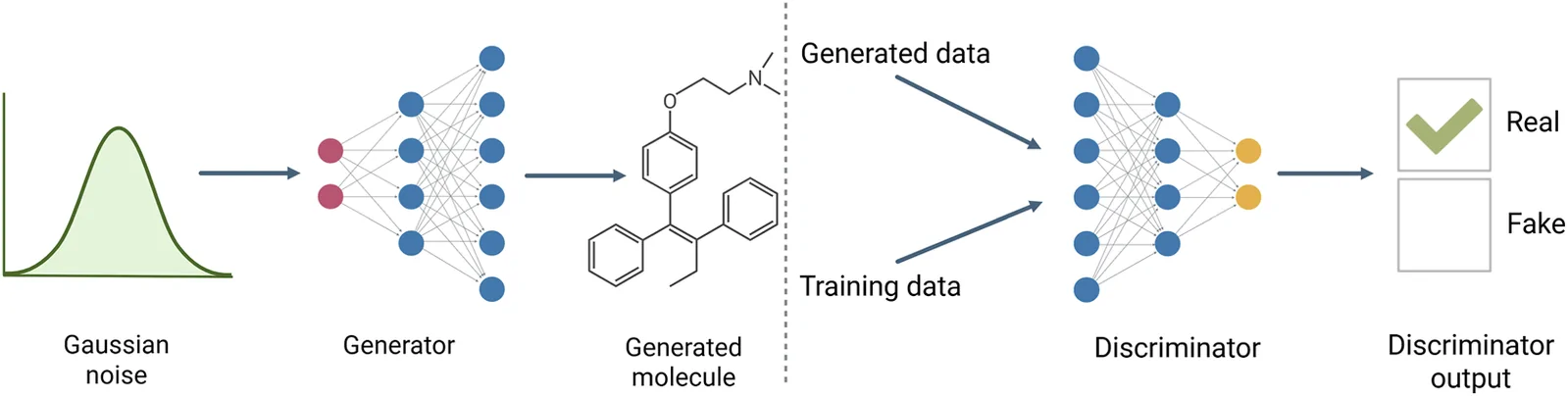
The adversarial framework of a generative adversarial network (GAN). A generator network synthesizes novel molecular configurations from random Gaussian noise. Simultaneously, a discriminator network evaluates these generated candidates against a distribution of real training data, providing iterative feedback to refine the generative output until it achieves high structural realism.

Generative Adversarial Networks (GANs) offer a highly dynamic framework for navigating expansive chemical spaces through a competitive learning paradigm (Gangwal et al., 2024). The architecture comprises two distinct neural networks—a generator and a discriminator—trained simultaneously (Figure 7). The generator synthesizes novel molecular configurations, while the discriminator evaluates them against a training distribution of established drugs to assess chemical realism. This adversarial process forces the model to iteratively refine its outputs, ultimately producing highly realistic, synthesizable compounds (Kotkondawar et al., 2025). Consequently, GANs excel at generating structurally distinct lead candidates optimized for specific pharmacological profiles, significantly accelerating therapeutic discovery (Pang et al., 2024; Farhadi et al., 2025).

Despite their transformative potential, standard GAN implementations often encounter bottlenecks in information retention and structural integrity, driving a shift toward more specialized frameworks. For instance, Zhang et al. proposed BD-CycleGAN (Zhang C. et al., 2024), which incorporates BiLSTM with a molecular cycle generative adversarial network to better preserve input information, resulting in enhanced molecular diversity and improved docking scores during structural transformation. Addressing the need for spatial context, Song et al. developed DNMG (Song et al., 2023), a Wasserstein-variant GAN that integrates 3D grid spatial information and atomic physicochemical properties. This model demonstrates superior binding affinity for specific targets, such as MK14 and CDK2, by effectively scanning the pharmacochemical space through an improved captioning network. To overcome the trade-off between property optimization and sample diversity, Macedo et al. introduced POWGAN (Macedo et al., 2025), a graph-based generator utilizing an adaptive reward-scaling policy. This architecture achieved 1.00 fully connected connectivity in quinoline-like molecules while simultaneously increasing the proportion of molecules with optimized lipophilicity (LogP) from 17% to 45%. Furthermore, Abbasi et al. engineered a feedback-driven GAN framework that incorporates an encoder-decoder and a non-dominated sorting genetic algorithm (Abbasi et al., 2022). This approach successfully reconstructed 99% of datasets including complex stereochemical information and achieved high internal and external diversity levels (0.88 and 0.94, respectively), effectively identifying novel lead candidates with high binding affinity for the Kappa Opioid and Adenosine A2a receptors. Together, these developments illustrate the evolution of GANs into robust, goal-directed platforms for high-throughput chemical exploration.

Such progress notwithstanding, the adversarial framework remains susceptible to training instabilities and the persistent threat of mode collapse, which can significantly restrict the diversity of the generated chemical library. A more fundamental challenge arises from the discrete nature of molecular representations, as standard GAN gradients are often incompatible with non-continuous data such as SMILES strings or graph indices. As a complementary optimization strategy, reinforcement learning (RL) supports goal-directed molecular generation and lead optimization by guiding sequential molecular modifications according to rewards related to chemical validity, drug-likeness, target affinity, and ADMET properties. Recent Deep Q-Network-based studies have demonstrated the ability of RL to generate highly valid drug-like molecules (Shakeri and Far, 2025). However, RL remains sensitive to reward design and may suffer from reward hacking, bias, and fairness concerns when surrogate objectives or training data are poorly specified (Shakeri et al., 2026). Addressing these generative and optimization limitations is therefore essential to ensure that AI-generated candidates are chemically valid, structurally diverse, biologically relevant, and suitable for downstream validation.

#### 4.2.7 Diffusion models

**FIGURE 8:**
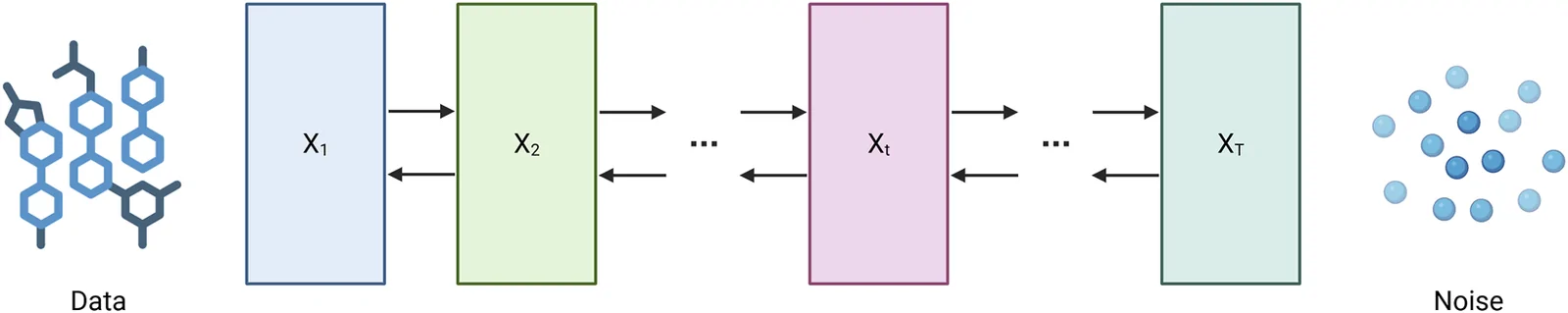
Workflow of a diffusion model. The architecture operates via a two-phase process: a forward diffusion phase (left to right) that progressively injects Gaussian noise into the original molecular data until it degrades into pure noise (XT), and a trained reverse denoising phase (right to left) that iteratively reconstructs valid, structured molecular conformations from the noise distribution.

Diffusion Models have emerged as a powerful class of generative frameworks (Alakhdar et al., 2024), operating through a two-step process: forward diffusion, which progressively adds Gaussian noise to molecular data, and reverse denoising, which learns to reconstruct the original signal (Figure 8). By iteratively refining structures from pure noise, these models achieve exceptional sample quality and diversity. In the context of drug discovery, diffusion-based approaches excel in 3D molecular generation and pocket-conditioned design (Sako et al., 2025). Their ability to capture complex spatial constraints and generate high-fidelity conformations makes them indispensable for structure-based drug design, where precise geometric arrangements are critical for binding affinity (Qian et al., 2023).

Advancing this paradigm, several high-performance frameworks have been developed to capture the intricate biophysical interactions within binding pockets. For instance, DiffBP addresses the limitations of sequential autoregressive generation by utilizing a non-autoregressive approach to denoise element types and 3D coordinates simultaneously, effectively modeling pair-coupled energy functions across entire molecules (Lin et al., 2025). To bridge the gap between local chemical motifs and global atomic structures, the AMDiff (Atom-Motif Consistency Diffusion Model) employs a hierarchical joint-training architecture; this multi-view learning strategy has demonstrated superior generative validity in case studies targeting protein kinases such as ALK and CDK4 (Li G. et al., 2025). To further refine target-awareness, BindDM adaptively extracts essential binding subcomplexes and processes them via SE(3)-equivariant networks, achieving a significant average Vina score of −5.92 on the CrossDocked2020 benchmark (Huang Z. et al., 2024). Beyond structural coordinates, the integration of multi-modal biological descriptors is exemplified by SurfDock, which incorporates surface-level features and optimizes molecular degrees of freedom on non-Euclidean manifolds (Cao et al., 2025). SurfDock’s practical utility was notably validated through the identification of seven novel hit molecules targeting aldehyde dehydrogenase 1B1 (ALDH1B1), underscoring the transformative potential of diffusion models in prospective virtual screening and reliable complex prediction. Furthermore, Xu et al. developed 3D-EdiffMG (Xu C. et al., 2025), a framework that enhances scaffold-based generation and lead optimization by utilizing an equivariant encoder based on dual strong and weak atomic interaction forces. By incorporating a gated multilayer perceptron block with tiny attention to capture long-range dependencies within macromolecules, this model has demonstrated significant utility in generating potent candidates against mutant strains of the Monkeypox virus using the tecovirimat scaffold, ensuring favorable ADMET profiles while maintaining structural integrity. DiffSBDD further demonstrated that a single SE(3)-equivariant, pocket-conditioned diffusion model can be reused for *de novo* generation, molecular inpainting and iterative property optimization without task-specific retraining, although its evaluations also revealed the overrepresentation of very small and very large ring systems (Schneuing et al., 2024). More recently, DiffGui introduced joint atom–bond diffusion and classifier-free guidance using affinity and drug-like property labels. On the PDBbind benchmark, it achieved higher atom stability, molecular stability, PoseBusters validity and RDKit validity than TargetDiff, DiffSBDD and other evaluated baselines, together with Vina Score, Vina Min and Vina Dock values of −6.700, −7.655 and −8.448, respectively; it also showed competitive generalization on CrossDocked and was evaluated in *de novo* design, lead optimization and wet-lab experiments (Hu et al., 2025). Collectively, these advancements signify a fundamental transition from stochastic molecular sampling toward a regime of biophysically-informed precision engineering. By synergizing multi-scale representations with equivariant generative dynamics, these frameworks provide a cohesive infrastructure capable of deciphering the non-linear intricacies of the protein–ligand interactome. The empirical success of these high-fidelity models in bridging the gap between *in silico* nomination and prospective experimental validation redefines the discovery landscape.

Such transformative potential notwithstanding, diffusion models are hampered by high computational costs because their iterative reverse-sampling is significantly slower than one-step generative architectures such as VAEs. A secondary challenge involves the difficulty of ensuring strict physical realism, such as bond length accuracy and atomic connectivity, within unconstrained 3D space. These efficiency bottlenecks underscore the urgent need for accelerated sampling methods to enable practical, high-throughput large-scale molecular design.

#### 4.2.8 Strategic rationale for architectural selection and synergistic integration

To synthesize these methodological advancements, Table 2 summarizes the core frameworks within the drug discovery pipeline. Admittedly, no single architecture provides a universal solution for complex therapeutic challenges; instead, modern strategies increasingly favor the synergistic integration of complementary models to process heterogeneous data modalities. From a task-specific perspective, CNNs efficiently extract local patterns from sequence, image, and voxelized inputs but provide limited global context and native geometric awareness. RNNs remain applicable to sequential molecular representations, although serial computation and long-range information decay restrict their scalability. Transformers offer stronger global-context modeling and benefit substantially from large-scale pre-training, which can improve adaptation to label-scarce downstream tasks, but their attention cost and data requirements remain considerable. GNNs are particularly suitable for molecular property prediction and interaction modeling because they directly encode atomic connectivity, although conventional variants often lack explicit three-dimensional spatial awareness. Generative frameworks such as VAEs, GANs, and diffusion models are frequently coupled with predictive modules to optimize pharmacochemical properties within three-dimensional conformational space. VAEs provide stable training, rapid decoding, and smooth latent-space optimization, and generally achieve high chemical validity when robust molecular representations and constrained decoders are used; however, reconstruction regularization may limit structural novelty and geometric fidelity. GANs support rapid generation of realistic, distribution-matched libraries, but adversarial instability, mode collapse, and difficulties in handling discrete molecular representations may reduce the validity and diversity of their outputs. Diffusion models offer stronger geometric control for pocket-conditioned and fragment-based 3D design and can achieve high structural validity through atom-, bond-, and geometry-aware denoising, although their outputs still require checks for valence compliance, molecular stability, and physical plausibility, while iterative sampling incurs substantially higher computational costs. Accordingly, VAEs are preferable for efficient latent-space exploration, GANs for rapid library generation when training stability can be controlled, and diffusion models for structure-based campaigns in which conformational accuracy outweighs computational latency. By leveraging these complementary strengths, researchers can reconcile computational efficiency, sequence context, molecular topology, and geometric precision. Strategic navigation of these architectural trade-offs, alongside multimodal integration, is essential for translating computational hits into viable therapeutic candidates.

## 5 Applications of deep learning in modern drug discovery

DL has profoundly transformed the pharmaceutical pipeline by accelerating each phase of therapeutic development. This chapter systematically explores the practical applications of these advanced computational models, covering target identification, virtual screening, *de novo* molecular design, retrosynthetic analysis, ADMET profiling, and intelligent drug repurposing.

### 5.1 Target identification and validation

Target identification and validation represent the critical yet costly inception of drug discovery, frequently hindered by systemic biological complexity (Masoomkhah et al., 2025). While conventional methods struggle with high-dimensional heterogeneous datasets, DL seamlessly integrates diverse multi-omics profiles (Bao et al., 2024; Ren et al., 2026). By autonomously extracting non-linear features, DL frameworks enable the highly precise identification of core therapeutic targets driving disease pathology (Lu Z. et al., 2025). In a notable application, Huang et al. developed MOGOLA (Huang et al., 2025), a graph-based DL framework designed for precise biomarker discovery. Applied to breast cancer datasets, MOGOLA performs end-to-end multi-omics integration without requiring manual feature selection. The model successfully identified biomarkers corresponding to clinically actionable pathways, thereby supporting subtype-specific interventions such as HER2-directed therapies. Furthermore, protein-protein interaction analysis within the framework pinpointed essential hub genes, including CCNA2, CDK1, and ESR1, which align robustly with established cancer hallmarks and known drug targets. This study clearly demonstrates how advanced graph architectures translate high-dimensional omics data into validated therapeutic vulnerabilities. Expanding the scope of DL-enabled target discovery beyond oncology to complex neurodegenerative disorders, Xu et al. introduced NETTAG (Xu et al., 2022), an interpretable framework designed to translate Genome-Wide Association Studies (GWAS) and multi-omics findings into actionable Alzheimer’s disease (AD) pathobiology. Their model predicted 156 AD-related genes (alzRGs), of which 38 encode proteins targeted by FDA-approved or investigational drugs. Crucially, the framework prioritized nine well-investigated AD therapeutic targets, including BACE1, CDK5, FYN, GSK3B, MARK4, MKL1, and PTK2B. By accurately linking specific drivers to disease hallmarks, such as connecting FYN and GSK3B to amyloid-β production and tau hyperphosphorylation respectively, this study not only validated canonical AD mechanisms but also provided a robust computational basis for drug repurposing strategies targeting the protein products of these newly identified alzRGs. Extending these computational capabilities to infectious diseases, Popov et al. proposed an integrated structural and DL framework to identify vulnerable drug binding sites within rapidly mutating viral proteins (Popov et al., 2023). By explicitly incorporating protein dynamics, spatial accessibility, and mutability, the model successfully mapped a conformation-specific, glycan-free binding site proximal to the receptor binding domain of the SARS-CoV-2 spike glycoprotein. Subsequent molecular dynamics simulations demonstrated that therapeutic targeting of this site allosterically prevents the critical closed-to-open conformational transition, effectively inhibiting interactions with the human angiotensin-converting enzyme 2 (ACE2) receptor. Furthermore, empirical validation via a pseudotyped virus assay confirmed multiple hit compounds capable of viral inhibition at micromolar concentrations, underscoring the capacity of DL to pinpoint and structurally validate viable therapeutic targets amid global health crises. Taken together, these advancements underscore a fundamental paradigm shift where DL serves as the primary engine for navigating the vast biological space to pinpoint and structurally validate viable therapeutic targets in modern drug discovery.

### 5.2 Virtual screening and hit discovery

Virtual screening constitutes a fundamental step in identifying active hit compounds from astronomically large chemical libraries, a process historically bottlenecked by the immense computational cost of physics-based molecular docking (Gentile et al., 2022; Zhang X. et al., 2024). While traditional screening methods struggle to evaluate massive chemical spaces efficiently, DL provides a rapid and data-driven alternative (Zhang et al., 2022; Joshi et al., 2023; Samad et al., 2023). By extracting robust topological and spatial features from molecular graphs and protein structures, DL architectures facilitate high-throughput binding affinity predictions, significantly accelerating the discovery of novel hit compounds (Jiang et al., 2022; Zhang H. et al., 2023; Dandibhotla et al., 2025). To overcome the generalization bottlenecks frequently encountered when deploying DL across vast and unfamiliar chemical spaces, Seo and Kim introduced PharmacoNet (Seo and Kim, 2024), a pioneering framework designed for ultra-large-scale virtual screening through DL-guided pharmacophore modeling. Diverging from standard approaches that directly estimate affinities from restricted training sets, PharmacoNet automates the extraction of protein-based pharmacophore features and evaluates ligand potency using a parameterized analytical scoring function. This strategic integration of DL with structured pharmacophore concepts ensures exceptional generalization capabilities across previously unseen targets and novel ligands, delivering an ultra-fast computational engine capable of reliably navigating massive chemical repositories. In a notable advancement for high-throughput hit discovery, Huang et al. developed MolTrans (Huang et al., 2021), an interaction Transformer framework designed to predict drug-target interactions (DTI) with high efficiency. To evaluate the practical efficacy of the MolTrans framework, Huang et al. conducted a targeted validation by retrieving test sets from the BIOSNAP database and mapping target proteins via GtoPdb. The model’s predictive robustness was rigorously benchmarked across four primary druggable categories, encompassing 1,908 interactions in enzymes, 533 in GPCRs, 496 in ion channels, and 104 in nuclear receptors. Further broadening the scope of virtual screening beyond canonical protein targets, Bae and Nam introduced DeepRNA-DTI (Bae and Nam, 2025), a sequence-based architecture tailored for the conventionally challenging domain of RNA-targeted therapeutics. The model demonstrated robust generalization across diverse RNA subtypes and was successfully applied to a high-throughput virtual screening of over 48 million compounds against oncogenic pre-miR-21. This large-scale application identified both established binders and novel chemical scaffolds with RNA-specific physicochemical properties, demonstrating how advanced DL frameworks can effectively expand the druggable genome by discovering promising RNA-targeting candidates from vast chemical spaces. Serving as a definitive testament to the empirical power of these DL frameworks, Liu et al. successfully translated computational virtual screening into a validated clinical asset by discovering a novel antibiotic against the multidrug-resistant pathogen *Acinetobacter* baumannii (Liu G. et al., 2023). Recognizing the high failure rates of conventional screening against this formidable Gram-negative bacterium, the researchers trained a neural network using an *in vitro* growth inhibition dataset comprising approximately 7,500 molecules. By deploying this trained model to rapidly explore vast, uncharted chemical spaces, they conducted highly targeted *in silico* predictions to identify structurally novel antibacterial agents. This DL-guided campaign yielded abaucin, a highly potent compound exhibiting narrow-spectrum activity. Subsequent empirical investigations elucidated its exact mechanism of action, confirming that abaucin perturbs lipoprotein trafficking by targeting the LolE protein. Crucially, the therapeutic efficacy of this computationally discovered hit was rigorously validated *in vivo*, successfully controlling A. baumannii infections in a murine wound model. Collectively, these milestones highlight the transformative power of DL, proving its capability to transition from theoretical computational models into tangible, life-saving clinical applications.

### 5.3 De novo molecular design and lead optimization

The transition from initial hits to optimized lead compounds requires navigating a vast chemical space to satisfy multiple conflicting pharmacological parameters (Li, 2023). While conventional medicinal chemistry relies heavily on iterative and human-guided modifications, generative DL models autonomously explore this multidimensional landscape (Khater et al., 2025). By leveraging architectures such as diffusion models and variational autoencoders, these frameworks generate novel chemical structures tailored to specific target pockets, enabling the simultaneous optimization of binding affinity and drug-like properties (He et al., 2023; Zhang Y. et al., 2023). Addressing the formidable challenge of multi-parameter optimization (MPO) in new chemical entity discovery, Perron et al. demonstrated the practical value of DL generative models within an active drug development campaign (Perron et al., 2022). Utilizing a sparse initial dataset of 881 molecules, the researchers trained predictive Quantitative Structure-Activity Relationship (QSAR) models across 11 distinct bioactivity criteria. By coupling these QSAR predictors with a ligand-based *de novo* design algorithm, they generated 150 virtual compounds exhibiting optimal *in silico* profiles. To empirically validate this computational pipeline, eleven candidates were synthesized and comprehensively tested against the target product profile. The AI-designed molecules achieved an impressive 86% success rate by meeting an average of 9.5 objectives, substantially outperforming the 58% success rate of compounds derived from traditional medicinal chemistry. Crucially, three of these novel molecules satisfied the stringent project requirements, including one compound that strictly met all 11 MPO objectives. This strategic structural innovation proved highly beneficial for navigating complex MPO constraints, ultimately highlighting the capacity of artificial intelligence to exploit uncharted and therapeutically viable chemical spaces. Advancing beyond ligand-based design, recent frameworks directly leverage 3D target structures to guide molecular generation. Lu et al. proposed DMDiff (Lu H. et al., 2025), a geometric spatial perception diffusion model that captures precise 3D spatial information to accurately design molecules with high binding affinities for specific protein targets. Building upon the potential of conditional diffusion, Huang et al. developed PMDM (Huang L. et al., 2024), an equivariant diffusion model incorporating both local and global molecular dynamics. PMDM facilitates highly efficient *in silico* generation and lead optimization by utilizing conditioned protein information without requiring task-specific retraining. To validate its translational readiness, the researchers conducted lead optimization for the SARS-CoV-2 main protease and Cyclin-dependent Kinase 2 (CDK2). *In vitro* evaluations of the synthesized lead compounds confirmed improved CDK2 activity alongside favorable selectivity profiles, proving that target-aware diffusion models can deliver tangible clinical assets. Furthermore, DL has revolutionized specific lead optimization operations, particularly fragment-based drug design. Igashov et al. introduced DiffLinker (Igashov et al., 2024), an equivariant 3D-conditional diffusion model engineered to generate synthetically accessible molecular linkers conditioned directly on target protein pockets. When tasked with connecting fragmented scaffolds, DiffLinker not only successfully recovered ground-truth structures but also identified over two hundred unique molecular topologies. This extensive exploration of the scaffold space highlights the ability of generative models to facilitate advanced scaffold hopping and fragment linking, thereby dramatically accelerating the transition from fragmented hits to highly optimized lead compounds.

### 5.4 Reaction prediction and retrosynthetic analysis

Ensuring the synthetic accessibility of novel therapeutic candidates remains a formidable challenge, often reliant on the intuition and manual experience of synthetic chemists (Long et al., 2025). While classical rule-based algorithms struggle with novel chemical transformations and rigid predefined templates, DL treats synthetic chemistry as a complex sequence translation task (Miller et al., 2025). By employing advanced sequence-to-sequence models and graph-based reasoning, DL frameworks accurately predict reaction outcomes and design efficient retrosynthetic routes, substantially reducing both the time and cost of chemical synthesis (Liu et al., 2024; Liu et al., 2026; Lan et al., 2025). To illustrate the practical utility of generative DL in complex retrosynthetic analysis, Chen et al. developed G2Retro (Chen et al., 2023), a two-step graph-based framework utilizing a semi-template approach for synthesis prediction. Demonstrating exceptional generalization, the model was evaluated on newly approved drugs, such as Mitapivat and Tapinarof, which were entirely absent from its training corpus. For Mitapivat, G2Retro correctly prioritized the exact amide coupling strategy reported in its discovery patent as the highest-ranked prediction, while autonomously generating diverse, chemically viable alternative routes, including sulfonamide formations utilizing various leaving groups. Similarly, for Tapinarof, the framework accurately identified critical deprotection strategies and proposed innovative carbon-carbon coupling mechanisms, including McMurry, Wittig, and Suzuki reactions. Furthermore, detailed analyses of additional test molecules confirmed G2Retro’s ability to discern multiple potential reaction centers within complex multi-amide scaffolds, distinguishing between highly efficient late-stage functionalizations and alternative sequential strategies. These findings underscore the capacity of graph generative models to not only accurately recover ground-truth synthetic pathways but also to autonomously propose highly diverse and chemically logical alternative routes, providing synthetic chemists with a comprehensive, data-driven engine for navigating complex molecular assembly. Building upon the success of graph-based methodologies, Chen and Jung proposed LocalRetro (Chen and Jung, 2021), a framework inspired by the chemical intuition that molecular transformations predominantly occur at localized reaction centers. Diverging from traditional approaches that rely on global structural analysis, LocalRetro employs a graph neural network to extract local atomic environments and predict reaction templates based on localized atom and bond modifications. Acknowledging that distal functional groups can secondarily influence reaction pathways, the researchers further integrated a global attention mechanism to account for nonlocal chemical effects. This sophisticated dual-level encoding enabled the model to achieve exceptional round-trip prediction accuracies of 89.5% and 87.0% on the USPTO-50K and USPTO-MIT benchmark datasets, respectively. Crucially, the practical applicability of LocalRetro was validated by successfully predicting the synthesis pathways of five distinct drug candidate molecules directly derived from established literature. Transitioning from graph topologies to sequence-to-sequence translation, Transformer architectures have also emerged as highly effective engines for template-free retrosynthetic planning. Wang et al. introduced RetroPrime (Wang X. et al., 2021), a versatile Transformer-based methodology that mirrors the sequential cognitive process of synthetic chemists. The model executes retrosynthesis in two distinct phases by first decomposing a target molecule into precursor synthons and subsequently generating complete reactants by attaching appropriate leaving groups. A persistent challenge in Transformer-based generation is the tendency to produce chemically implausible or insufficiently diverse outputs. RetroPrime specifically mitigates these defects by optimizing the generation strategy, ultimately achieving robust predictive performance comparable to state-of-the-art models on large-scale datasets such as USPTO-full. By simultaneously addressing the issues of diversity and chemical plausibility, sequence-based frameworks like RetroPrime offer a highly adaptable alternative to rigid template-matching algorithms, further expanding the computational toolkit available for complex organic synthesis.

Direct numerical comparison among retrosynthesis models requires caution. Although G2Retro, RetroPrime, and GTA were evaluated on USPTO-50K, their results were reported by independent studies and do not constitute a strictly controlled head-to-head comparison. Under settings without reaction-class information, the reported Top-1 accuracies were 53.9% for G2Retro, 51.4% for RetroPrime, and 51.1% for GTA. LocalRetro reported round-trip accuracy, which is not directly comparable with the exact-match Top-1 accuracies reported for these models. Moreover, RadicalRetro was evaluated primarily on the specialized RadicalDB and should therefore be interpreted within the context of radical retrosynthesis. Collectively, G2Retro, RetroPrime, and GTA showed broadly comparable reported Top-1 performance under settings without reaction-class information, whereas LocalRetro and RadicalRetro should be interpreted within their respective metric and reaction-domain contexts.

### 5.5 ADMET profiling and toxicity prediction

Comprehensive evaluation of absorption, distribution, metabolism, excretion, and toxicity (ADMET) profiles is imperative to mitigate the high attrition rates observed in late-stage clinical trials (Xiong et al., 2021). While conventional computational models and *in vivo* assays often fail to capture complex pharmacokinetic behaviors accurately, DL excels at mapping intricate structure-property relationships (Beckers et al., 2024). By autonomously extracting hidden chemical representations, DL models provide highly accurate and scalable predictions of systemic toxicity and metabolic stability, thereby de-risking candidates early in the developmental pipeline (Wu et al., 2023; Wang et al., 2024). Demonstrating the power of hybrid architectures in this domain, Shao et al. proposed an evolved Transformer model that enhances the standard Graphormer framework by integrating molecular fingerprints and physicochemical properties through a tree-based approach (Shao et al., 2024). This integration yielded state-of-the-art predictive capabilities on established ADME/Tox benchmarks, achieving a Pearson Correlation Coefficient of 0.86 while significantly improving mechanistic interpretability. Similarly addressing the need for comprehensive profiling, Myung and colleagues introduced Deep-PK (Myung et al., 2024), a robust platform leveraging graph neural networks and graph-based signatures. This framework successfully models 73 distinct endpoints, encompassing 64 specific ADMET properties, providing researchers with an interpretable computational engine to guide molecular optimization. Focusing explicitly on severe safety liabilities, Saravanan et al. developed a multi-model Graph Convolutional Network (GCN) approach tailored to diverse toxicity endpoints (Saravanan et al., 2024). By utilizing distinct training strategies for regression and binary classification tasks, the models achieved impressive predictive metrics across critical safety parameters, including hERG cardiotoxicity, mutagenicity, and hepatotoxicity. The researchers further integrated these GCN models into a virtual screening pipeline using strict thresholds derived from approved drugs, demonstrating a highly effective strategy for filtering out toxic compounds during early discovery. Finally, to facilitate high-throughput decision support, Fu et al. developed ADMETlab 3.0 (Fu et al., 2024), an updated predictive platform utilizing a multi-task Directed Message Passing Neural Network (DMPNN) coupled with molecular descriptors. This architecture ensures rapid calculation speeds across numerous endpoints while maintaining superior robustness. Crucially, the inclusion of uncertainty estimates within the prediction outputs provides medicinal chemists with a rigorous statistical basis for confidently prioritizing candidate compounds prior to extensive experimental validation. Ultimately, these sophisticated predictive methodologies transform ADMET profiling from a reactive late-stage hurdle into a proactive data-driven strategy, fundamentally reshaping the entire drug discovery pipeline toward the efficient development of safer and more efficacious clinical candidates.

### 5.6 Drug repurposing

Drug repurposing offers a highly efficient strategy to bypass the prolonged timelines and safety risks associated with *de novo* compound development (Fiscon et al., 2021). While traditional repositioning discoveries have largely been serendipitous, DL enables the systematic identification of new therapeutic indications for existing approved drugs (Wang F. et al., 2022). By integrating complex heterogeneous data types, including biomedical knowledge graphs and transcriptomic perturbation signatures, DL frameworks accurately map novel drug-disease associations, unlocking alternative treatment avenues for emerging diseases with remarkable efficiency (Hsieh et al., 2021; Yue and Dutta, 2025). Capitalizing on the predictive accuracy of structural representation learning, Yasir et al. deployed a Graph Convolutional Network (GCN) via the DeepChem framework to repurpose FDA-approved drugs as novel JAK2 inhibitors (Yasir et al., 2024). By extracting complex three-dimensional molecular features to predict binding affinities, the DL model successfully identified several non-canonical candidates, including ribociclib, topiroxostat, amodiaquine, and gefitinib. Crucially, these computational predictions were rigorously confirmed through subsequent molecular docking and *in vitro* biological validation, proving that these compounds exhibit robust JAK2 inhibitory activity comparable to the established standard, tofacitinib. This study exemplifies how GCNs can surpass traditional screening paradigms to unveil hidden therapeutic potential within existing pharmacopeias. Shifting the computational focus from single-target interventions to sophisticated combinatorial therapies, Li et al. proposed DeepDrug (Li V. O. K. et al., 2025), an expert-guided artificial intelligence methodology tailored for Alzheimer’s Disease (AD). Rather than targeting an isolated mechanism, this framework utilizes Graph Neural Networks to encode a highly complex, weighted biomedical graph encompassing somatic mutations, aging pathways, and immunological markers. By systematically capturing granular relationships within this new embedding space, DeepDrug effectively prioritized a synergistic five-drug combination, consisting of tofacitinib, niraparib, baricitinib, empagliflozin, and doxercalciferol. This multi-target approach concurrently modulates neuroinflammation, mitochondrial dysfunction, and glucose metabolism, showcasing the unparalleled capacity of DL to orchestrate high-order combinatorial drug repurposing. Beyond chronic and neurodegenerative conditions, the rapid adaptability of DL repositioning frameworks proved indispensable during acute global health crises, such as the COVID-19 pandemic. Deepthi et al. engineered an efficacious DL ensemble model specifically to prioritize clinically validated antiviral agents against the SARS-CoV-2 pathogen (Deepthi et al., 2021). By integrating structural chemical similarities with viral genome sequences to generate robust feature vectors, the Convolutional Neural Network accurately inferred potential drug-virus associations. Extensive case studies corroborated the model’s empirical credibility, as top-ranked predictions, including ribavirin, nitazoxanide, and favipiravir, were subsequently validated by clinical literature and advanced into active human trials. These collective advancements unequivocally demonstrate that DL-driven drug repurposing has evolved into a highly robust, scalable, and indispensable strategy for modern therapeutic discovery.

### 5.7 Summary of applications and case studies

The practical utility and transformative potential of DL in this field are exemplified by the diverse range of successful applications summarized in Table 1. The strategic deployment of advanced neural architectures has fundamentally transformed critical stages of pharmaceutical research, spanning from initial target identification and high-throughput virtual screening to *de novo* molecular design, retrosynthetic analysis, and precise ADMET profiling. By systematically integrating multimodal biological and chemical data, these predictive and generative frameworks substantially accelerate therapeutic discovery. Nevertheless, Table 1 should be interpreted as a structured cross-study synthesis rather than a direct ranking of model performance. Because the included studies differ in datasets, data splits, endpoint definitions, auxiliary information, and evaluation metrics, their reported results cannot be reliably aggregated into a single normalized score. Accordingly, meaningful comparisons should be restricted to task- and benchmark-matched settings, while definitive head-to-head conclusions require standardized preprocessing, identical data splits, and unified evaluation pipelines.

## 6 Challenges and future perspectives

Despite the remarkable progress of DL in modern drug discovery, several critical challenges continue to hinder its widespread and reliable application. These challenges are not only technical but also conceptual, spanning issues related to data quality, model interpretability, experimental validation, and the escalating intensity of computational resource requirements. Addressing these multidimensional limitations will be essential for translating DL-based discoveries into clinically actionable therapeutics while maintaining an inclusive and equitable research environment. In addition to discussing these field-level challenges, this section also reflects on the scope and limitations of the present review and outlines priorities for future research.

### 6.1 Data quality and scarcity

The efficacy and generalizability of DL models in drug discovery remain fundamentally constrained by noisy, heterogeneous, and incompletely labeled datasets. Experimental inconsistencies and the preferential reporting of successful results can introduce systematic bias, while the scarcity of reliable negative samples may cause models to misclassify untested drug–target pairs and overestimate predictive performance (Kumar et al., 2022). Addressing these limitations requires rigorous data curation together with transfer learning and federated learning (FL) to exploit large-scale molecular knowledge and distributed proprietary datasets (Huang D. et al., 2024). Transfer learning enables representations learned from extensive chemical libraries to be adapted to data-limited tasks, whereas FL supports collaborative model training without centralizing confidential data. The MELLODDY project demonstrated this potential by securely aggregating model updates across ten pharmaceutical companies while retaining data within each partner’s infrastructure (Oldenhof et al., 2023). In parallel, high-quality negative samples should be selected rather than assuming that all unknown interactions are inactive; network-based selection strategies can reduce hidden sampling bias and improve DTI prediction robustness (Cheng et al., 2025). Collectively, these strategies provide a practical route toward more reliable and transferable DL models under realistic data constraints.

### 6.2 Interpretability and the black box problem

Despite the impressive predictive accuracy of DL architectures, their inherent “black box” nature remains a critical bottleneck in AI-driven drug discovery. The structural complexity of these models often prioritizes high-dimensional pattern recognition over mechanistic transparency, obscuring the biological logic behind specific predictions. This opacity fosters a significant trust crisis between computational systems and drug discovery practitioners, as the inability to provide verifiable biological justifications hampers the integration of AI outputs into high-stakes clinical and regulatory workflows. To mitigate this skepticism, the field must transition toward explainable artificial intelligence (XAI) frameworks (Ding et al., 2025). Distinguished from standard interpretability that relies on statistical correlations, causality-aware XAI identifies the specific structural determinants governing biological responses through interventional or counterfactual reasoning. By isolating true causal drivers from spurious correlations, researchers can strategically modulate pharmacophores with higher confidence, ensuring that computational insights align with the underlying biophysical mechanisms of action (Li et al., 2024; Shi et al., 2025).

### 6.3 Experimental validation and translational bottlenecks

Even when DL models generate promising predictions, translating these computational insights into experimentally validated therapeutics remains a substantial challenge. Many AI-driven studies report high predictive performance on benchmark datasets but lack systematic wet-lab validation, limiting their real-world impact (Liu D. et al., 2025). Biological systems are inherently complex, and *in silico* predictions often fail to account for dynamic factors such as cellular context, metabolic stability, and systemic toxicity profiles. This disconnect creates a translational bottleneck where the rapid generation of virtual candidates far outpaces the current throughput of experimental testing. Bridging this gap requires the adoption of closed-loop autonomous laboratories that orchestrate an iterative Design-Make-Test-Analyze (DMTA) cycle (Brocklehurst et al., 2024; Li J. et al., 2025). In this paradigm, generative models function as orchestrators for robotic synthesis platforms and automated bioassays, where experimental results are immediately fed back into the model to refine the chemical search space in real-time. A concrete implementation was demonstrated by Dai et al. (2024), who integrated mobile robots with an automated synthesis platform, liquid chromatography–mass spectrometry, and benchtop nuclear magnetic resonance spectroscopy in a modular autonomous laboratory. Nevertheless, achieving full autonomy necessitates the development of standardized interoperability protocols and robust error-handling frameworks to manage the stochastic variability of chemical reactions. By transforming discovery into a self-optimizing engine, these closed-loop systems can drastically reduce the timelines and financial burdens associated with therapeutic validation.

### 6.4 Computational resource intensity and accessibility

The transition toward large-scale foundation models and massive pre-training on billions of molecular entities has introduced an exponential escalation in computational requirements. The training of such massive architectures demands unprecedented high-performance computing infrastructure, drastically driving up research and development expenditures and capital investment. To mitigate these operational burdens, the field is increasingly prioritizing algorithmic efficiency through techniques such as knowledge distillation and model compression. Knowledge distillation specifically attenuates computational overhead by transferring the predictive power of a massive teacher model into a compact student architecture without a substantial loss in accuracy (Sheshanarayana and You, 2025). Additionally, model quantization and architectural pruning reduce the memory footprint for deployment on more accessible hardware (Rasool et al., 2025). By emphasizing efficiency over mere computational scale, the research community can democratize access to cutting-edge tools, ensuring that therapeutic innovation remains driven by biophysical insight rather than raw processing power.

### 6.5 Limitations of the present review and future research directions

Several limitations should be acknowledged. Given the breadth and rapid evolution of AI-driven drug discovery, this review focuses on representative and methodologically influential studies, and some newly emerging models or highly specialized applications may not have been fully captured. Cross-study comparisons are also constrained by substantial heterogeneity in datasets, preprocessing procedures, data splits, endpoints, baseline models, and evaluation metrics. Moreover, much of the available evidence remains based on retrospective benchmarks and computational validation, with limited prospective experimental or clinical confirmation. Future studies should establish standardized, task-specific benchmarks, transparent reporting practices, external validation, uncertainty estimation, and fair head-to-head comparisons under consistent conditions. Greater integration of multimodal data, physics-informed modeling, causality-aware explainable AI, computationally efficient architectures, and closed-loop experimental platforms will be important for improving reproducibility, practical reliability, and translational relevance.

## 7 Conclusion

In this review, we have systematically summarized the transformative role of DL in modern drug discovery, spanning from foundational molecular representation learning to advanced neural architectures and their diverse applications across the pharmaceutical pipeline. By integrating heterogeneous biomedical data, DL frameworks have significantly enhanced key stages including target identification, virtual screening, *de novo* molecular design, retrosynthetic analysis, ADMET profiling, and drug repurposing, thereby reshaping traditional discovery paradigms into a more data-driven and autonomous process. Collectively, the rapid evolution of DL architectures, including convolutional and recurrent networks, transformers, graph neural networks, and especially advanced generative models, has enabled the extraction of complex and high-dimensional patterns that were previously inaccessible. These generative and predictive advancements have not only improved property accuracy but have also vastly expanded the navigable chemical and biological space, thereby facilitating the discovery of novel therapeutic opportunities across multiple disease domains.

Despite this progress, clinical translation remains bottlenecked by several critical challenges. First, data quality and scarcity, particularly experimental noise and negative data deficits, limit model generalization, necessitating federated and transfer learning to leverage decentralized data. Second, the opacity of “black-box” models hinders clinical trust, warranting a pivot toward causality-aware Explainable AI to translate statistical correlations into verifiable biological mechanisms. Furthermore, to bridge the disconnect between *in silico* computational design and empirical validation, integrating computational pipelines with closed-loop autonomous laboratories is essential for iterative, real-world feedback. Finally, the prohibitive computational overhead associated with large-scale models necessitates a strategic shift toward algorithmic efficiency and model compression, ensuring that cutting-edge AI tools remain accessible to the broader scientific and medicinal community. Taken together, no single DL architecture is universally superior; model suitability depends on the alignment among molecular representation, task requirements, available data, and validation context. By linking architectural capabilities with the strengths and limitations of reported validation strategies, this review provides a decision-oriented basis for distinguishing computational promise from experimentally supported translational potential. Ultimately, overcoming the remaining barriers will transform artificial intelligence from a predictive computational strategy into a practical, end-to-end ecosystem, accelerating the delivery of safe and efficacious therapeutics.
